# Nonlinear memristive computational spectrometer

**DOI:** 10.1038/s41377-024-01703-y

**Published:** 2025-01-14

**Authors:** Xin Li, Jie Wang, Feilong Yu, Jin Chen, Xiaoshuang Chen, Wei Lu, Guanhai Li

**Affiliations:** 1 State Key Laboratory of Infrared Physics, Shanghai Institute of Technical Physics, Chinese Academy of Sciences, 500 Yu-Tian Road, Shanghai, 200083 China; 2 School of Physical Science and Technology, ShanghaiTech University, Shanghai, 201210 China; 3 University of Chinese Academy of Science, No. 19A Yuquan Road, Beijing, 100049 China; 4 Hangzhou Institute for Advanced Study, University of Chinese Academy of Sciences, No.1 Sub-Lane Xiangshan, Hangzhou, 310024 China; 5 Shanghai Research Center for Quantum Sciences, 99 Xiupu Road, Shanghai, 201315 China

**Keywords:** Spectrophotometry, Optoelectronic devices and components

## Abstract

In the domain of spectroscopy, miniaturization efforts often face significant challenges, particularly in achieving high spectral resolution and precise construction. Here, we introduce a computational spectrometer powered by a nonlinear photonic memristor with a WSe_2_ homojunction. This approach overcomes traditional limitations, such as constrained Fermi level tunability, persistent dark current, and limited photoresponse dimensionality through dynamic energy band modulation driven by palladium (Pd) ion migration. The critical role of Pd ion migration is thoroughly supported by first-principles calculations, numerical simulations, and experimental verification, demonstrating its effectiveness in enhancing device performance. Additionally, we integrate this dynamic modulation with a specialized nonlinear neural network tailored to address the memristor’s inherent nonlinear photoresponse. This combination enables our spectrometer to achieve an exceptional peak wavelength accuracy of 0.18 nm and a spectral resolution of 2 nm within the 630–640 nm range. This development marks a significant advancement in the creation of compact, high-efficiency spectroscopic instruments and offers a versatile platform for applications across diverse material systems.

## Introduction

Spectrometers are pivotal in material characterization, industrial testing^1,2^, and image sensing, fueled by the growing demand for devices that are portable, accurate, and offer superior spectral resolution and broad bandwidth^3–10^. However, the conventional spectrometer design, which relies on bulky optical components and lengthy optical paths, clashes with the push towards miniaturization, creating a notable obstacle to compact device development^2,11^.

Efforts to achieve miniaturization have led to the exploration of photonic crystals, metasurfaces, and compact interferometry as alternatives to traditional dispersive elements^12–20^. Despite these advancements, miniaturizing spectrometer components typically compromises resolution, dynamic range, or signal-to-noise ratios, highlighting a fundamental trade-off in spectrometer design: compactness often diminishes performance^21–26^.

The emergence of two-dimensional (2D) materials presents a promising pathway to surmount these challenges due to their atomic-scale tunability and strong light-matter interactions^27–32^. Miniature computational spectrometers leveraging 2D materials utilize advanced algorithms to enhance spectral resolution and reconstruction accuracy without degrading device performance^33–35^. Nonetheless, these solutions face limitations, including restricted Fermi level tunability due to gate voltage modulation, challenges in dark current suppression, and constraints on the photoresponse matrix dimensionality arising from the linear relationship between photogenerated current and incident light, which collectively hinder accurate, high-resolution spectral analysis^36–41^.

In this work, photonic memristors^42–47^ emerge as a groundbreaking alternative, utilizing their distinctive energy band coordination and memory effects for the high-performance spectrometer design. By leveraging the nonlinear properties of memristors, we introduce a Positive-Intrinsic-Negative (PIN) WSe_2_ homojunction-based nonlinear spectrometer that offers high performance and resolution within an ultra-compact footprint. This device surpasses conventional van der Waals heterojunctions in quantum efficiency and introduces a neural network-based spectral prediction system, combining theoretical and empirical data for precise, robust, and broadband spectral reconstruction. Our work not only sets a new standard in spectrometry by achieving high accuracy and resolution in a compact form but also offers a scalable solution for diverse material systems.

## Results

**Fig. 1:**
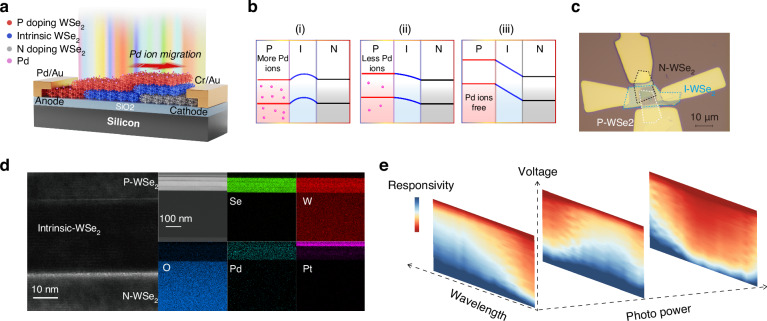
Architectural and Functional Overview of the Nonlinear Photonic Memristor-Based Microspectrometer. **a** Schematic of the device showing a homogeneous PIN configuration with a p-doped WSe_2_ layer incorporating Pd ions for enhanced memristive functionality. **b** Illustration of energy band evolution in different resistance states due to Pd ion migration, demonstrating the device’s band structure adjustability. **c** Optical image of the device with a 10 μm scale bar. **d** Cross-sectional transmission electron microscopy (TEM) image of the PIN WSe_2_ structure, displaying the layers of P- WSe_2_, intrinsic- WSe_2_, and N- WSe_2_ with a 10 nm scale bar (left). The energy-dispersive X-ray spectroscopy (EDS) spectrum on the right shows the elemental composition, including W, Se, O, Pd, and Pt, with a 100 nm scale bar (right). **e** The measured nonlinear optical response, illustrating the enhancement beyond traditional photoresponse matrices by adding a third dimension

Our miniaturized computational spectrometer lies in the nonlinear responses to stimuli wavelength and optical power, facilitated by bias voltages across varying resistance states. As shown in Fig. 1a, the device’s architecture features a nonlinear, homogeneous PIN photonic memristor, consisting of sequentially layered P-doped, intrinsic, and N-doped WSe_2_. The device is a two-terminal device with Pd as the anode and Cr/Au as the cathode. The device fabrication process is elaborated in Supplementary Fig. S1. Notably, the integration of defects at the Pd/WSe_2_ interface enhances Pd ion migration and relaxation within the P-type WSe_2_ layer under external voltage, promoting ionic movement. We achieve remarkable control over the device’s band structure via Pd ion modulation, as illustrated in Fig. 1b. This process, which allows for the alteration of the top WSe_2_ layer’s polarity, offers advantages over conventional gate voltage modulation by providing a wider adjustment range, simpler methodology, and more precise targeting. Figure 1c presents an optical microscopy image of our device. The transfer sequence of the WSe_2_ layers is N-doped, intrinsic, and P-doped WSe_2_. The transfer curves confirming the doping types are shown in Supplementary Fig. S2.

Figure 1d combines a TEM image with elemental analysis, highlighting the high-quality interfaces between WSe_2_ layers and metal contacts, essential for efficient carrier flow across the channel. These interfaces remain largely unaffected by surface states from defects or vacancies. The corresponding EDS results validate the effective integration of Pd ions into the channel. It is worth noting that the device preparation process does not involve platinum; the platinum element is due to the standardized protection layer deposited via sputtering and a carbon protective layer prior to TEM testing. The detailed process is described in the Methods part.

Figure 1e showcases the measured photoresponse of our photonic memristor. The device’s nonlinearity arises from unique Pd ion migration and relaxation dynamics, which significantly differ from previously reported devices^29–31^. Nonlinear regression analysis, based on variable power responsivity matrices, underscores the device’s robust nonlinear behavior under diverse illumination scenarios. Exposed to an unknown spectrum, the device delineates a photocurrent versus an applied two-terminal voltage profile.

**Fig. 2:**
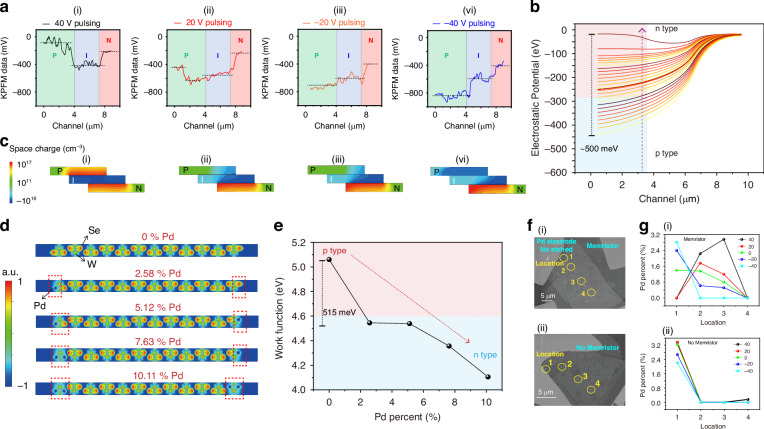
Energy Band Evolution and Nonlinear Spectral Characteristics in the Photonic Memristor. **a** Normalized relative potential distribution across the device’s transport channel, shown via Kelvin Probe Force Microscopy (KPFM) measurements. Sequences i-iv demonstrate the memristor’s state initialization at various voltage pulses from 40 V to -40V, revealing potential redistribution. **b** Simulated potential evolution within the device, showcasing the extensive modulation capability as the top WSe_2_ layer transitions from p-type doping at 10^16^ cm^−3^ to n-type doping at 10^17^ cm^−3^. **c** TCAD simulation illustrating the evolution of polarity within the space charge region, transitioning from n-doped at 40 V pulsing to p-doped at -40V pulsing, thereby demonstrating the adaptability of the top p-type WSe_2_ layer. **d** Local charge density in WSe_2_, with Pd ion doping concentrations set at 0%, 2.58%, 5.12%, 7.63%, and 10.11%, respectively. **e** Variation in work function as a function of doping concentration. **f** Scanning Electron Microscope (SEM) images of the device (**i**) with and (**ii**) without the memristor structure. Channel numbers are labeled inside. **g** Distribution of Pd ions across channels 1, 2, 3, and 4 of the memristor (**i**) and non-memristor device (**ii**) after preprocessing with voltage pulses at 40 V, 20 V, -20V, and -40V

Our study of the photonic memristor’s band evolution, as illustrated in Fig. 2a, identifies a critical transition in surface potential distribution from highly-doped n-type to highly-doped p-type behavior within the top p-layer, induced by sweeping the voltage stimulus from 40 V to −40V. This band tuning mechanism diverges from traditional gate-voltage controlled devices by enabling a wider array of energy band modifications, further detailed in Supplementary Fig. S3. Whereas conventional gate-controlled devices modulate interface barrier height to adjust doping concentration and spectral responsivity, our memristive device fundamentally alters material polarity, significantly broadening the energy band adjustment range. Remarkably, our device’s Fermi energy level tunability approaches E_g_, surpassing conventional adjustments limited to within E_g_/2. To investigate the Pd ion migration mechanism, we employed TCAD simulations to visualize the transition from N-doping to P-doping in the top p-type WSe_2_ layer. As shown in Fig. 2b, the potential distribution across the three regions of the device is depicted. This distribution aligns with the surface potential variation range of approximately 500 meV for p-type WSe_2_, as measured by KPFM. In Fig. 2c, these simulations elucidate the evolution of the spatial charge region, demonstrating its effect on device polarity and energy band structure. Further details are provided in Supplementary Fig. S4 and Supplementary Note 1.

It is noteworthy that the introduction of Pd ions, indicated by surface potential changes, leads to n-doping of WSe_2_. To further understand the internal doping mechanism, we calculated the kinetic process of in vivo migratory Pd ion doping in WSe_2_ using the Vienna Ab initio Simulation Package (VASP), see Supplementary Note 2 for modeling details. Figure 2d illustrates a notable decrease in charge density around Pd, suggesting that Pd acts as a donor, thereby converting WSe_2_ to an n-type semiconductor. Additionally, Pd’s strong Coulomb repulsion and its lowered binding energy with W indicate that only a minimal driving force is required to release Pd, which aligns with our experimental observations. We calculated the local charge densities for Pd ion doping concentrations of 0%, 2.58%, 5.12%, 7.63%, and 10.11%. The results show that Pd replaces Se with a surrounding charge that exhibits a dominant energy level state. Subsequently, we calculated the figure of merit for these five different Pd ion doping concentrations, as shown in Fig. 2e. It is evident that the work function of the material gradually decreases as the Pd ion doping concentration increases, further supporting the conclusion that Pd ion migration induces the transition of WSe_2_ to n-type.

To experimentally confirm the role of Pd ions in this polarity transition, we fabricated a comparative device with a buffer layer by etching P-WSe_2_ with SF_6_ and O_2_ to prevent Pd ion penetration into the channel. Figure 2f(i) presents an SEM image of the device exhibiting memristive properties (without etching during the process). We tested the concentration of Pd ions at four points—1, 2, 3, and 4—with point 1 being closest to one end of the Pd electrode. When pre-treated with -40 V, Pd ions congregated near the Pd electrode. As the pre-treatment voltage increased, Pd ions gradually diffused into the channel, reaching a maximum concentration at location 3, as shown in Fig. 2g(i). This provides direct evidence of the correlation between Pd ion concentration and pretreatment voltage.

Figure 2f(ii) shows the device without memristive behavior (processed with etching). In this case, no Pd ion migration occurred after pretreatment with different voltages, as shown in Fig. 2g(ii). Notably, the maximum Pd ion concentration measured in the channel was 2.94%, and the range of variation in the WSe_2_ surface potential, as measured by KPFM, was approximately 500 meV. This is consistent with VASP calculations, which indicate that a doping concentration of 2.58% results in a work function change of 515 meV.

**Fig. 3:**
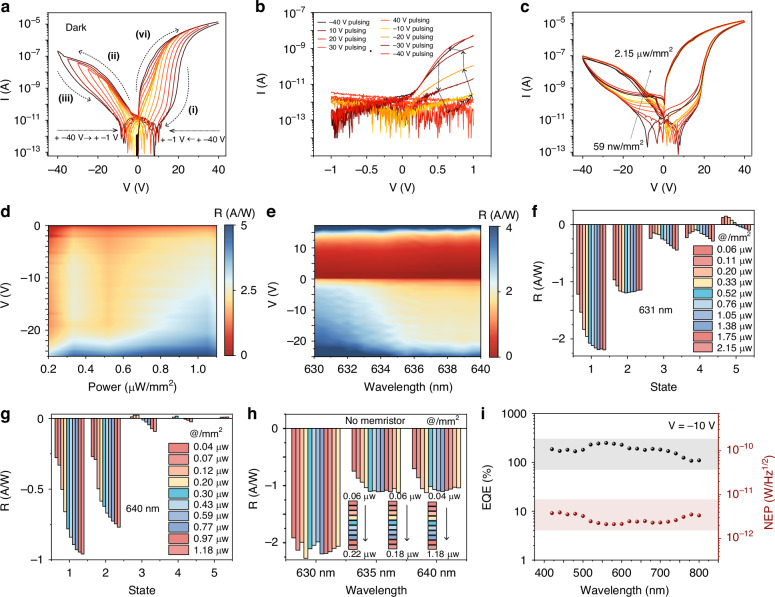
Nonlinear Photoresponse of the Photonic Memristor to Incident Light Powers. **a** Dark current response curves to different initial voltages. The nonlinear and memristive response through sweeping voltage loops is labeled i to iv. **b** Dark current variations with V ranging from −1V to +1 V after a high-voltage cycle from −40V to +40 V and back. **c** Displays the device’s nonlinear photocurrents under various optical stimuli, captured across a scanning voltage loop from 40 V to −40V. **d** The photoresponse to 631 nm light at different power levels and biases. The device showcases sensitivity to both light intensity and applied voltage. **e** Photoresponse over a range of wavelengths and voltages at a constant incident power. **f–g** The memristive photoresponses to monochromatic light at 631 nm and 640 nm under five different initial bias conditions. States are identified as 1 (−40 V), 2 (10 V), 3 (20 V), 4 (30 V), and 5 (40 V), each applied for 20 seconds during initial preprocessing. **h** Illustrates the photoresponses after etching the top WSe_2_ layer to introduce a barrier against Pd ion migration. It effectively diminishes the memristive characteristics. This panel also compares the linear photoresponse behaviors of the modified (non-memristive) device post-chemical processing, using 631 nm, 635 nm, and 640 nm monochromatic light as stimuli. **i** Noise-equivalent power (NEP) and external quantum efficiency (EQE) of the device at −10V

To investigate the effect of Pd ion migration on the carrier transport process, we tested the dark current with different starting voltages, as shown in Fig. 3a. The amnesic current intensity as a function of applied voltage (IV) is divided into four processes (i), (ii), (iii), (vi), and the whole process shows a ‘butterfly shape’ with different starting voltages. As shown in Fig. 3b, we show the non-volatile nature of this amnesia device. It can be seen that at different pulse voltages, the voltage of device IV changes from a positive conduction state to a cut-off state and then back to a conduction state as the pulse voltage changes.

Figure 3c details the effect of palladium ion migration on the energy band structure of our device in the presence of light. It showcases the device’s nonlinear photoresponse to 631 nm irradiation at diverse power levels, where the photocurrent output curves reveal a distinct nonlinear growth pattern. This pattern emerges directly from the redistribution of Pd ions within the transport channel, triggered by the variance in light irradiation power levels. These variances prompt internal ion movement, leading to hysteresis or advancement effects that disrupt the band structure alignment, culminating in the observed photocurrent nonlinearity. In Fig. 3d, we unveil the changes in responsivity across power and voltage spectra, marking a significant departure from the behavior of conventional gate-voltage-modulated devices. Our device stands out by maintaining a dark current in the picoampere range from −20 V to 20 V, thereby improving spectral discrimination accuracy and enabling the detection of narrow spectra. The distinctive nonlinear light response across different wavelengths is further investigated in Fig. 3e. Here, the dynamic modulation of the energy band structure by Pd ions across various memristor states is demonstrated, resulting in noticeable spectral response nonlinearity (additional details in Supplementary Figs. S5-S8).

Figure 3f and g detail the device’s sensitive response to light at 631 nm and 640 nm under varying voltage conditions, elucidating its complex dynamics. The current-time charts (refer to Supplementary Fig. S9) and the depiction of device states under different initialization voltages provide insights into these dynamics. States 1 through 5 correspond to pre-test voltages of −40V, 10 V, 20 V, 30 V, and 40 V, each applied for 20 seconds. Comparative experiments, such as etching the top P-type WSe_2_ layer for 2 seconds (further information in Supplementary Fig. S10), exhibited reduced nonlinear responses, especially post-etching (Fig. 3h). This suggests that WO_X_ formation during etching hinders Pd ion integration with SF_6_ and O_2_, acting as a barrier to Pd ion transport. Further elucidation is provided in Supplementary Fig. S11. The observed reduction in pre-processing adaptability, as shown in Supplementary Figs. S12 and S13, underscores the pivotal role of Pd ions in ensuring the device’s operational efficacy. Responsivity comparisons, as outlined in Supplementary Fig. S14, are consistent with anticipated outcomes. The external quantum efficiency and noise equivalent power of the device were calculated for different wavelengths at a −10 V bias. The noise currents are detailed in Supplementary Fig. S15. The EQE is determined by the formula ${EQE}=\frac{{hcR}}{q\lambda }$, and NEP is calculated as ${NEP}=\frac{{I}_{{noise}}}{R(\lambda )}$. The EQE is approximately 200% within the 550-650 nm range, and the NEP is around 1 pW, as shown in Fig. 3i. The corresponding laser power density of the NEP, considering the device area, is 28 mW/m², which is significantly lower than the 6–9 W/m² power density of an LED lamb.

**Fig. 4:**
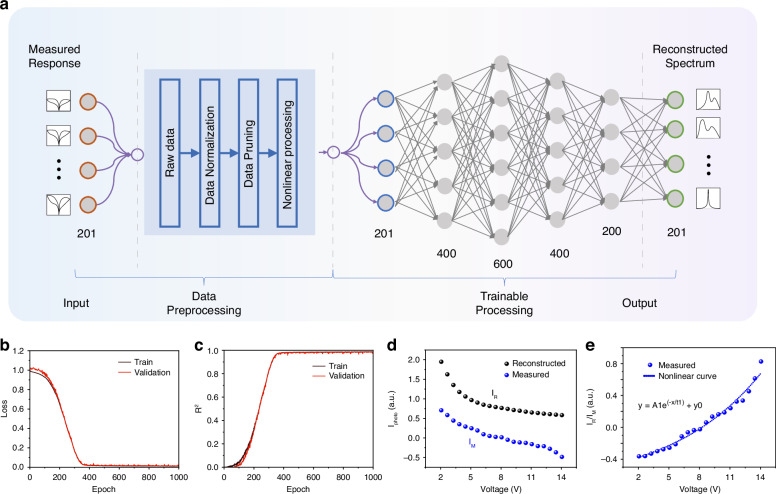
Construction of the Nonlinear Neural Network. **a** The process of spectral reconstruction: an unknown spectrum is incident on the photonic memristor, generating a nonlinear photoresponse. This data undergoes nonlinear regression preprocessing before being input into a custom-built nonlinear neural network for final spectrum prediction. **b** Loss curves for the training and validation sets. **c** Coefficient of determination (R^2^) for the training and validation sets, indicating the model’s fit. **d** The discrepancy between the photocurrent reconstructed from the spectral response matrix for narrowband stimuli and the measured reference photocurrent. **e** Introduction of a nonlinear regression curve, derived from the discrepancies between reconstructed and reference photocurrents. These values serve as nonlinear weighting factors to specifically enhance predictions during subsequent neural network processing

The unique ability of our photonic memristor to extensively modulate the energy band presents a groundbreaking nonlinear photoresponse, transforming spectral reconstruction methodologies. As illustrated in Fig. 4a, we demonstrate the spectral reconstruction of an unknown spectrum using the photonic memristor in combination with a custom-built neural network. This process is divided into four stages: Input, Data Preprocessing, Trainable Processing, and Output. It’s important to note that the predicted data is not included in the training data (Training data = training set (80%) + validation set (20%)). Once the network is trained, we can reconstruct the spectrum by simply measuring the photocurrent of the device when illuminated by the unknown spectrum. The photocurrent and spectral data are real response data obtained from our experiments, and the optical path for photocurrent data acquisition is shown in Supplementary Fig. S16.

The neural network is based on a fully connected architecture, where the number of nodes in the input layer corresponds to the number of sampling points of the photocurrent. The hidden layer consists of four layers with 400, 600, 400, and 200 nodes, respectively, each utilizing ReLU as the activation function. The output layer’s node count matches the number of sampling points of the output spectral data, and no activation function is used in the output layer. We employed the Adam optimizer and added L2 regularization to the model to prevent overfitting. The mean square error (MSE) was used as the loss function, and the training process was conducted over 1000 epochs. The neural network model was designed to handle the nonlinear nature of our device’s spectral response, which traditional computational methods struggle to manage effectively. Traditional methods, such as photoresponse matrix derivation, are not feasible for handling the complexity of our four-dimensional (I-P-V-λ) photocurrent dataset. Therefore, employing a neural network is both reasonable and necessary to achieve accurate spectral reconstruction. As detailed in Supplementary Fig. S17, this approach allows us to efficiently train nonlinear neural network models for spectral reconstruction, demonstrating good generalization on a test set.

During the training process, as shown in Fig. 4b, the loss in both the training and validation sets decreases with the number of iterations, indicating continuous model optimization. The consistent decrease in validation loss, along with the training loss, suggests that our model is improving its prediction performance on new data, demonstrating strong generalization capabilities. The fact that the loss values in the validation set are not significantly different from those in the training set and decrease steadily indicates that our model is effectively adapting to and handling unseen data, thereby enhancing the reliability of the model in practical applications. Continuing training after the loss has stabilized helps fine-tune the model parameters, ensuring stable performance and minimizing short-term fluctuations.

To assess the regression model’s performance during training, we use the coefficient of determination (R^2^) as a statistical measure. The formula for R^2^ is ${{\rm{R}}}^{2}=1-\frac{{\rm{SSE}}}{{\rm{SST}}}$, where SSE (Sum of Squares due to Error) measures the total deviation of the predicted values from the actual data, and SST (Total Sum of Squares) represents the total variance in the actual data. As shown in Fig. 4c, the R^2^ value gradually approaches 1 as the number of training iterations increases. This convergence indicates that the model’s predictions are becoming increasingly accurate and closely fitting the actual data. The continuous improvement in the R^2^ value demonstrates the model’s effective learning and optimization, leading to more precise spectral data reconstruction.

A key challenge in predicting complex spectra is the amplification of electrical signals between wavelengths due to the nonlinear spectral response, which becomes more pronounced as the overall power increases. The limited amount of data may not suffice for accurate prediction of complex spectra. To mitigate this issue, we introduce a nonlinear preprocessing method to reduce the impact of nonlinearity. A notable difference between the computed photocurrent (I_R_) and the actual measured photocurrent (I_M_) is highlighted in Fig. 4d. To address this variance, we employ nonlinear weighting factors to devise a regression equation, displayed in Fig. 4e. This equation, integrating nonlinear fitting with neural network training parameters, introduces a preprocessing stage to reduce uncertainties caused by nonlinear responsivity, thereby improving prediction precision.

**Fig. 5:**
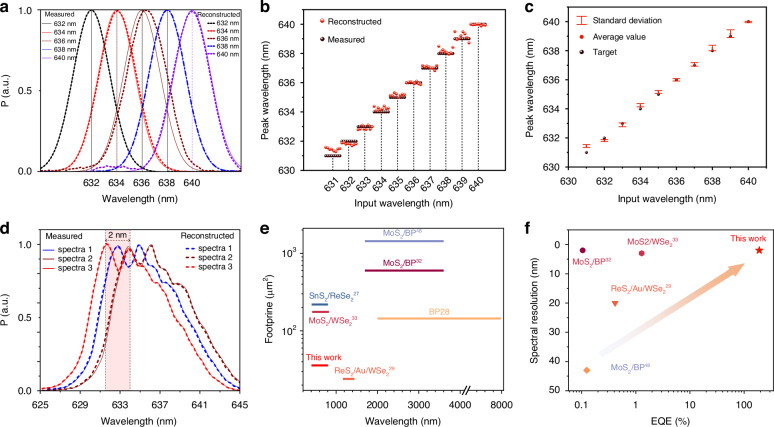
Spectral Reconstruction with the Nonlinear Memristive Spectrometer. **a** Comparison of the spectra reconstructed by the nonlinear neural network (dashed line) against measured reference spectra (solid line), each with a bandwidth of 2 nm. **b** Prediction results for the center wavelength across multiple reconstructed spectra. **c** Standard deviation and error in the prediction results, with an average difference of 0.18 nm in peak wavelength for all reconstructions. **d** Prediction of complex spectra, incorporating the nonlinear regression curve into the neural network (solid lines for measured reference spectra and dashed lines for reconstructed spectra). **e** Comparison of various miniaturized spectrometers utilizing bandgap engineering for spectral reconstruction. The horizontal axis represents the response bandwidth, while the vertical axis represents device size^27–29,32,33,48^. **f** Performance comparison of different bandgap-engineered, reconfigurable miniature spectrometers. The horizontal axis represents external quantum efficiency, and the vertical axis represents spectral resolution^29,32,33,48^

Once the network was constructed, we evaluated the spectrometer. This spectrometer accurately predicts the monochromatic spectrum over a 2 nm half-width as shown in Fig. 5a. To thoroughly assess the performance of this nonlinear computational spectrometer, we retested eight sets of photocurrents at wavelengths ranging from 631 to 640 nm as inputs. For a precise evaluation of the spectral prediction performance, we extracted and compared the peak wavelengths of the reconstructed spectra with the target values. The final prediction results are illustrated in Fig. 5b. We analyzed the distribution of these peak wavelength predictions and their corresponding target values. As shown in Fig. 5c, the mean and standard deviation of the prediction errors are presented, revealing an average difference of 0.18 nm between the predicted and target peak wavelengths within the 631–640 nm range. It is important to note that the dynamic adjustment of the energy band, influenced by palladium ion migration, may impact the stability and reproducibility of the device’s performance. To mitigate this, we implemented a zeroing calibration method: after each data acquisition, a + 40 V pulse voltage is applied for 20 seconds to ensure Pd ions are at one end of the P-doped WSe_2_ electrode before each scan. Dark current repeatability is detailed in Supplementary Fig. S18, and photoelectric current repeatability in Supplementary Fig. S19. To assess the repeatability of the fabrication process, we tested additional devices, which exhibited consistent memristive behavior and similar electrical characteristics as shown in Supplementary Fig. 20. These findings underscore the stability and repeatability of our PIN WSe_2_-based photonic memristor, making it a potential component for spectroscopic applications. As to the stability and repeatability of our device, we conducted a series of experiments to ensure its robustness under varying conditions. Variable temperature tests revealed that the overall current of the device increases with rising temperatures due to the elevated intrinsic carrier concentration. Additionally, the memristive effect, driven by Pd ion migration, became more pronounced at higher temperatures, confirming the reliability of the ion migration mechanism. Environmental robustness was further demonstrated through pressure variation tests, where the dark current remained consistent across a range of pressures from 1.013 × 10^3^ mbar to 10^−3^ mbar. Moreover, long-term stability was validated by monitoring the dark current over a period of 10 days, with no significant degradation observed as shown in Supplementary Fig. 21.

Figure 5d demonstrates the ability of the model to predict complex spectra, proving the ability of the spectrometer to reconstruct peak spacing spectra down to 2 nm. To further validate our device’s performance, we compared its size and reconstruction bandwidth with several typical miniature spectrometers, as shown in Fig. 5e. It is evident that our device offers a more compact form factor compared to other miniature spectrometers. In the context of spectrometer performance, the device’s responsivity and quantum efficiency are crucial factors that directly influence the accuracy of spectral discrimination (narrower spectra require lower optical power). Notably, while most current miniature spectrometers rely on heterojunctions for spectral reconstruction, these heterojunctions often suffer from potential barriers at the junction region due to energy band differences, leading to losses in optical response. One of the key advantages of our device, which utilizes homogeneous junctions, is the avoidance of such potential barriers, thus preserving the device’s optical response. As illustrated in Fig. 5f, our device achieves an external quantum efficiency of up to ~200% and demonstrates a high-precision spectral identification accuracy of 0.18 nm. Additionally, we successfully reconstructed a broad spectrum ranging from 400 to 800 nm using the same method, as shown in Supplementary Figs. S22 and S23, further demonstrating the universality and feasibility of our approach. Our research underscores the exceptional compactness and efficiency of our nonlinear miniature spectrometer, as highlighted in Supplementary Table S1, setting a new benchmark for device miniaturization in spectral analysis.

## Discussion

Our work represents a groundbreaking advancement in spectral analysis, unveiling the integration of photonic memristors with an advanced neural network to achieve spectral reconstruction. This spectrometer lies in the single photonic memristor’s capacity for extensive energy band modulation through Pd ion migration, heralding a new dimension of nonlinear photoresponses. This advancement not only outstrips the capabilities of traditional gate-voltage modulated devices in dynamic range and tuning precision but also pioneers a method for the accurate reconstruction of unknown spectra. Our research introduces a nonlinear miniature spectrometer that marries the unique attributes of photonic memristors with the computational power of neural networks, thereby redefining benchmarks for spectral resolution, accuracy, and miniaturization. The successful prediction of complex spectra with exceptional precision, alongside a novel approach to spectral reconstruction, marks a significant leap forward in spectral sensing technology. Beyond providing a novel instrument, our work paves the way for future explorations and developments in the domain of photonic memristor-based technologies and computational spectrometry.

## Materials and methods

### Device Fabrication

Our process begins with the mechanical exfoliation of few-layer WSe_2_ from commercial bulk crystals, procured from Shanghai Onway Technology Co., Ltd. The N-doped WSe_2_ was prepared by doping with rhenium (Re), and the P-doped WSe_2_ was prepared by doping with niobium (Nb). These layers are then transferred onto a silicon substrate, which is coated with a 285 nm silica layer. The thickness of the WSe_2_ layers is meticulously assessed using an optical microscope, ensuring precision in subsequent steps. The fabrication of Pd electrodes involves a series of steps: spin-coating photoresist, conducting photolithography, followed by coating and stripping processes. A similar procedure is employed for the creation of Cr electrodes. It is pertinent to note that non-memristive structures necessitate etching prior to the deposition of Pd. The intricate details of our fabrication process are elaborately documented in Supplementary Figures S1 and S10.

### Device characterization

The morphology and dimensions of the exfoliated WSe_2_ flakes and the completed devices are verified using a Zeiss Lab5 optical microscope. Electrical characterizations are conducted with a Keithley 4200 parameter analyzer, equipped with a 4200-PA remote preamplifier module. For assessing the thickness and surface potential, we employ scanning probe microscopy (Bruker Multimode III SPM), with KPFM measurements performed using n-doped silicon tips in tapping mode at zero bias. The spectral data essential for our neural network training is acquired through a commercial spectrometer and monochromator (Zolix), supplemented with lasers (OYSL). TEM samples are prepared using a Thermo Scientific Helios G4 HX dual-beam system, safeguarded by a platinum layer deposited via sputtering and a carbon protective layer. TEM imaging is executed in a Thermo Scientific Tecnai F20 microscope (200 kV), complemented by element distribution analysis through EDS mapping.

### Spectral reconstruction

#### Single-peak spectral prediction

We trained the neural network using known photocurrents and corresponding spectra as input and output, respectively. The trained network was then applied to reconstruct unknown spectra not included in the training set.

#### Source data

We selected the 631 nm to 640 nm wavelength band for spectral reconstruction, using 1 nm increments. For each wavelength, 10 different power levels were chosen, and 5 sets of tests were conducted under consistent conditions. To enhance the dataset, we reorganized the photocurrent data and introduced noise to simulate measurement errors, resulting in a comprehensive dataset of 10 wavelengths × 10 power levels × 10 variations × 5 repetitions = 5000 data points. It is important to note that the predicted data were not included in the training data (Training data = training set (80%) + validation set (20%)). Consequently, the training data comprised 5000 data points, with the training set containing 4000 points and the validation set containing 1000 points. Each set of photocurrents consisted of 201 points, with each current value corresponding to an energy band in the memristive state of the device, resulting in a total of 20,100 energy band states utilized in the training process.

#### Complex spectra prediction

Before training, we computed the photocurrent *I*_*i*_ for the composite spectrum based on the single-peak basis vector responsivity and calculated the ratio of *I*_*i*_ to the test photocurrent to account for non-linear factors. This ratio represents the computational error due to non-linearities. Multiple datasets were used for training. The test photocurrents were then multiplied by the nonlinear regression equation, which served as input for spectral reconstruction.

## Supplementary information

Supplementary information

## Data Availability

Relevant data supporting the key findings of this study are available in the article and Supplementary Information file. All raw data generated in this study are available from the corresponding author upon reasonable request.

## Code availability

The code used for data analysis during this study is available upon reasonable request from the corresponding authors.
