# Supplementary material for: Nonlinear memristive computational spectrometer
Source: Light Sci Appl. 2025 Jan 14;14:47. doi: 10.1038/s41377-024-01703-y (PMC11733003; doi:10.1038/s41377-024-01703-y)
Supplement: Supplementary file 1 — Supplementary information [file 41377_2024_1703_MOESM1_ESM.docx]

**Supplementary Information for**

**Nonlinear Memristive Computational Spectrometer**

**Xin Li^1,2,3^, Jie Wang^1^, Feilong Yu^1^, Jin Chen^1^, Xiaoshuang Chen^1,3,4,5^, Wei Lu^1,2,3,4,5^, Guanhai Li^1,3,4,5*^**

^1^State Key Laboratory of Infrared Physics, Shanghai Institute of Technical Physics, Chinese Academy of Sciences, 500 Yu-Tian Road, Shanghai, 200083, China

^2^School of Physical Science and Technology, ShanghaiTech University, Shanghai 201210, China

^3^University of Chinese Academy of Science, No. 19A Yuquan Road, Beijing 100049, China

^4^Hangzhou Institute for Advanced Study, University of Chinese Academy of Sciences, No.1 Sub-Lane Xiangshan, Hangzhou, 310024, China

^5^Shanghai Research Center for Quantum Sciences, 99 Xiupu Road, Shanghai, 201315, China

^*^ ghli0120@mail.sitp.ac.cn;

**Table of contents**

**Note 1. Calculation of Band Structure and Electrical Transport Properties of PIN WSe_2_.**

**Note 2. The Calculated Doping Effect of Pd ion on WSe_2_ Energy Band with VASP.**

**Fig. S1. Stepwise Fabrication of the Nonlinear Microspectrometer.**

**Fig. S2. Transfer Curves for Different Doping WSe_2_ Devices.**

**Fig. S3. Comparison of Energy Band Modulation: Gate-Control vs. Ion Migration.**

**Fig. S4. TCAD Simulation of Nonlinear Photonic Memristor Dynamics.**

**Fig. S5. Diverse Photocurrent Responses across Wavelengths for Spectral 1 to Spectrum 5.**

**Fig. S6. Diverse Photocurrent Responses across Wavelengths for Spectral 6 to Spectrum 10.**

**Fig. S7. Measured Spectral Power for Spectrum 1 to Spectrum 10.**

**Fig. S8. Partial Dynamic Nonlinear Response of the Memristor Across Power, Voltage, and Wavelengths.**

**Fig. S9.** **Nonlinear Response of Memristor under 631nm Irradiation.**

**Fig. S10. Process for Altered Memristive Behavior in PIN Devices.**

**Fig. S11.** **Photo- and Dark- Current Response in Non-Memristive Devices under Monochromatic Light.**

**Fig. S12. Output Characteristic Curves of Non-Memristor under Voltage Scanning.**

**Fig. S13. Dark Current Behavior in Non-Memristor Devices across Various States.**

**Fig. S14. Responsivity Comparison between Memristor and Non-Memristor Devices.**

**Fig. S15: Noise Currents at Different Operating Voltages.**

**Fig. S16. Spectrometer Measurement Optical Setup.**

**Fig. S17. Spectral Reconstruction via Nonlinear Neural Networks.**

**Fig. S18. Dark Current Repeatability Property of the Fabricated Memristors.**

**Fig. S19. Photocurrent Repeatability** **of the Fabricated Memristors.**

**Fig. S20. Dark IV Curves of the** **Fabricated Multiple Devices.**

**Fig. S21. The Environment Robustness Evaluation of the Fabricated Device.**

**Fig. S22. Reconstruction of Single Peak Spectrum with Narrow Linewidth across 400-800 nm.**

**Fig. S23. Reconstructed versus Actual Spectra for Large Bandwidth across 400nm-800 nm.**

**Table S1. Performance Comparison with Reported Computational Spectrometer.**

**Supplementary Note 1. Calculation of Band Structure and Electrical Transport Properties of PIN WSe_2_.**

The TCAD simulations in our paper are designed to verify the modulation of the WSe_2_ energy band by Pd ions. The process includes three steps: device modeling, material parameter preparation, and physical model application.

1. **Device Modeling:**
2. All three WSe_2_ layers are designed to be 30 nm thick.
3. The N-type WSe_2_ doping concentration is 10^17^ cm^-3^, and the intrinsic doping concentration is 10^15^ cm^-3^. The P-type doping concentration varies from P-doped 10^17^ cm^-3^ to N-doped 10^16^ cm^-3^ in gradients to characterize the effect of Pd ion migration on the energy bands.
4. The structural grid density is set to 0.5 nm×0.05 nm (non-junction region) and 0.005 nm×0.005 nm (junction region).
5. **Energy Band Structure Calculation:**
6. Carrier concentration versus Fermi energy level and carrier mobility are calculated using standard semiconductor equations.
7. The basic transport model of WSe_2_ involves solving the Poisson equation and continuity equations for electrons and holes.
8. Key parameters in the WSe_2_ parameter file include bandgap, effective density of states, mobility, and recombination models.
9. Here are the equations and key parameters using the calculations.

For nondegenerate n-type semiconductor (E_C_-E_F_ > 2k_B_T)

| $N_{n}=N_{c}exp(-\frac{E_{c}-E_{v}}{k_{B}T})$ |  |
| --- | --- |

For degenerate n-type semiconductor (E_C_-E_F_ ≤ 2k_B_T)

| $N_{n}=N_{c}\frac{2}{\sqrt{\pi}}F_{1/2}(-\frac{E_{c}-E_{v}}{k_{B}T})$ |  |
| --- | --- |

$N_{c}=2\times{(\frac{m_{e}^{*}k_{B}T}{2\piћ^{2}})}^{\frac{3}{2}}={K(\frac{m_{e}^{*}}{m_{0}})}^{\frac{3}{2}}$ (K=2.5094×10^19^) is the conduction band equivalent density of states, $k_{B}$ is the Boltzmann constant, $T$ is the temperature,$m_{e}^{*}$ is the electron effective mass^1^, and $F_{1/2}$ is the Fermi integral function. Mobility is calculated as^2^$\mu=\frac{I_{\mathrm{ds}}L}{V_{\mathrm{ds}}n_{2D}}$. Finally, the electron mobility and hole mobility were calculated as 13 cm^2^V^−1^s^−1^ and 1 cm^2^V^−1^s^−1^, respectively^3^.

The basic transport model of WSe_2_ is

| $\nabla^{2}\varphi=-\frac{q}{\varepsilon}(N+p-n)$ |  |
| --- | --- |

Here $\varphi$ is the potential, $N$ is the net doping concentration, $p$ is the electron concentration, and $n$ is the hole concentration.

| $\frac{\partial n}{\partial t}=\frac{1}{q}\nabla\cdot\vec{J_{n}}+(G-R)$ |  |
| --- | --- |

Eq. 4 implies the electron continuity, where G and R denote the carrier production rate and the complex rate, respectively.

| $\frac{\partial p}{\partial t}=-\frac{1}{q}\nabla\cdot\vec{J_{p}}+(G-R)$ |  |
| --- | --- |

Eq. 5 is the hole continuity equation, where G and R denote the carrier production rate and the complex rate, respectively.

| $\vec{J_{n}}=qD_{n}\nabla n-nq\mu_{e}\nabla\varphi$ |  |
| --- | --- |

Eq. 6 is the electron current transport equation, including diffusion and drift currents.

| $\vec{J_{p}}=-qD_{p}\nabla p-pq\mu_{p}\nabla\varphi$ |  |
| --- | --- |

Eq. 7 is the hole current transport equation, including diffusion and drift currents.

See the table below for key parameters in the WSe_2_ parameter file:

| Bandgap | eDOSMass | hDOSMass | Epsilon | Scharfetter | ConstantMobility |
| --- | --- | --- | --- | --- | --- |
| Chi0= 3.7  Eg0= 1.63  Tpar=3.0000e+02  alpha = 0.0  beta= 0.0 | Formula=2  Nc300=3.415e+17 | Formula=2  Nv300=3.2368e+17 | epsilon=  3.9 | taumax=  1e-9, 1e-9  Nref=1e+16, 1e+16  gamma= 1, 1  Talpha= 0, 0  Tcoeff= 0, 0  Etrap= 0 | mumax=  0.133, 0.1  Exponent=  0.55846,  0.4298 |

1. **Physical Modeling:**

Mobility models, intrinsic density models, and recombination models are applied to simulate device behavior accurately.

**Supplementary Note 2.** **The Calculated Doping Effect of Pd ion on WSe_2_ Energy Band with VASP.**

We utilized VASP based on density-functional theory (DFT) to investigate the electronic properties of WSe_2_ and its doping by Pd at the Se-site.

1. **Calculation Setup:**
2. Perdew-Burke-Ernzerhof (PBE) functional in the generalized gradient approximation (GGA) is used for exchange-correlation interactions.
3. A 350 eV energy cutoff and a 15 × 15 × 1 Monkhorst-Pack k-point grid ensure convergence.
4. The energy convergence criterion is set to 10^-5^ eV atom^-1^, and the structural relaxation criterion is a Hellmann-Feynman force of 0.01 eV Å^-1^.
5. **Model Building:**
6. Geometry optimization is performed to determine equilibrium lattice constants and atomic positions.
7. Self-consistent electronic structure calculations follow until the total energy variation is less than 10^-6^ eV atom^-1^.


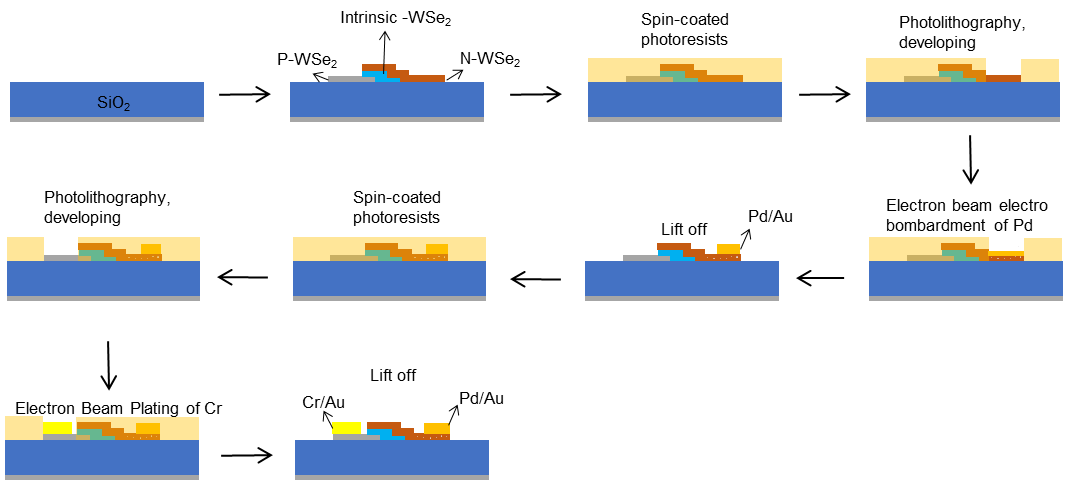


**Fig. S1.** **Stepwise Fabrication of the Nonlinear Microspectrometer.** This figure outlines the meticulous process used to create the device, starting with the mechanical exfoliation of WSe_2_ flakes from bulk crystals. These flakes are then transferred onto a SiO_2_/Si substrate to form a PIN structure. A photoresist layer is applied to the WSe_2_ surface before defining the anode pattern via laser direct writing lithography. High-speed Pd deposition (30 nm) follows, utilizing an electron beam evaporator to establish the anode. The process continues with the detachment of the anode end, and the creation of the drain end involves spin-coating another layer of photoresist, additional lithography, and a final detachment step, culminating in a device with Cr/Au (5 nm/25 nm) serving as the cathode.


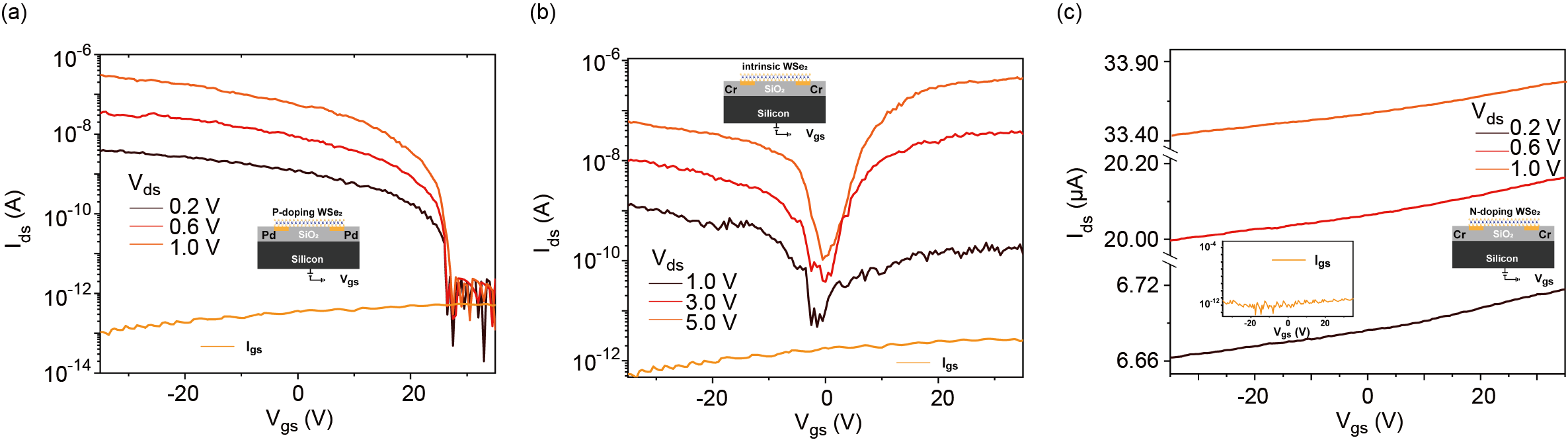


**Fig. S2. Transfer curves for different doping WSe_2_ Devices.** Transfer characteristics of (a) P-doped WSe_2_ (Nb-doped), (b) intrinsic WSe_2_, and (c) N-doped WSe_2_ (Re-doped). The insets show schematic diagrams of the corresponding field-effect transistors for each device. The transfer curves clearly demonstrate the carrier transport behavior specific to each material.


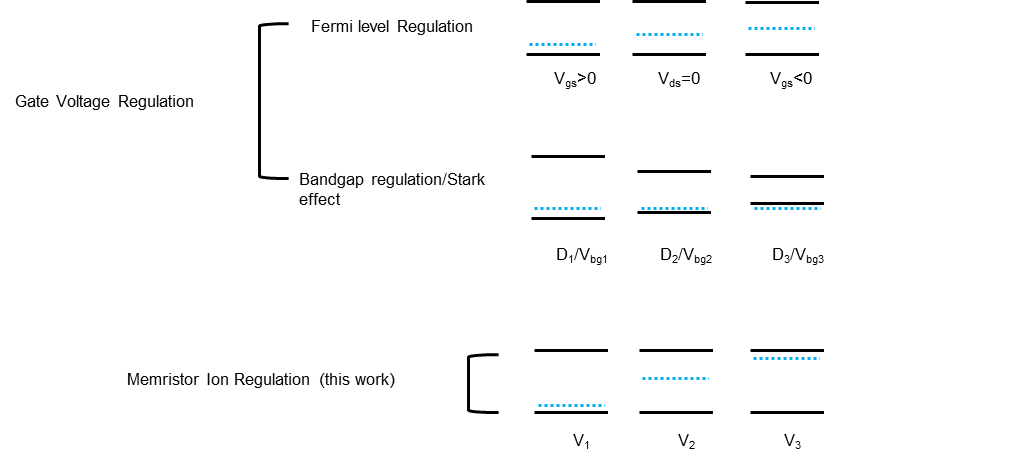


**Fig. S3:** **Comparison of Energy Band Modulation: Gate-Control vs. Ion Migration.** This figure contrasts the traditional gate voltage modulation of energy bands, which is typically limited to Fermi level modulation in unipolar materials and bandgap width modulation (or Stark effect), with the advanced technique of ion migration. Gate-controlled modulation is constrained in its ability to adjust the Fermi level and can only alter the bandgap width within a specific range. In contrast, the innovative approach of amnesia tuning through ion scanning significantly extends the modulation capacity, enabling dramatic adjustments to the material's Fermi level.


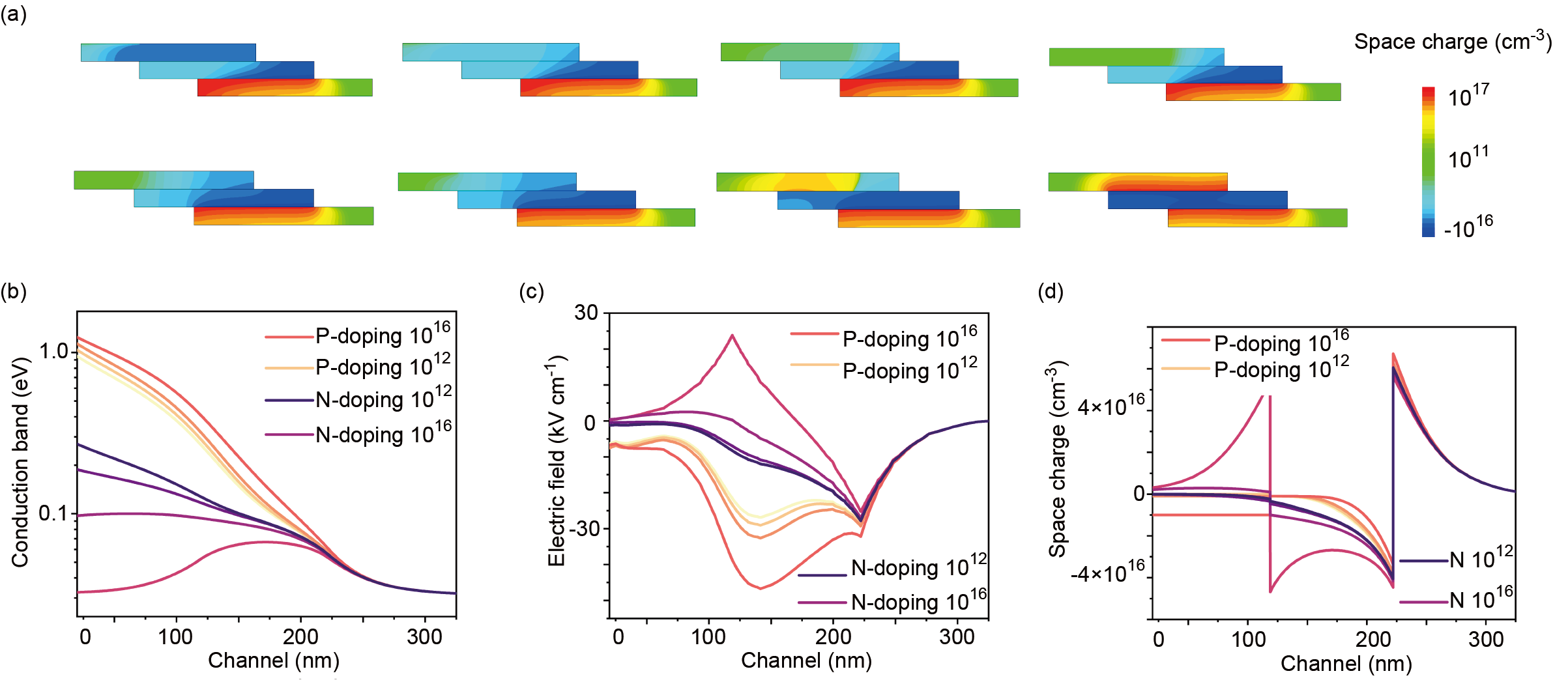


**Fig. S4:** **TCAD Simulation of Nonlinear Photonic Memristor Dynamics.** (a) Illustrates the space charge region's evolution in TCAD simulations, transitioning from highly doped P (-40 V pulsing) to highly doped N (40 V pulsing), depicted from left to right. (b) Shows the pin band diagram's evolution in the P layer, moving from a highly doped P state to a highly doped N state with voltage pulsing. (c) Depicts the distribution of the built-in electric field within the pin structure as it transitions from P to N doping states. (d) Visualizes the spatial charge concentration distribution within the pin structure, highlighting the significant modulation from P to N doping states due to voltage pulsing.


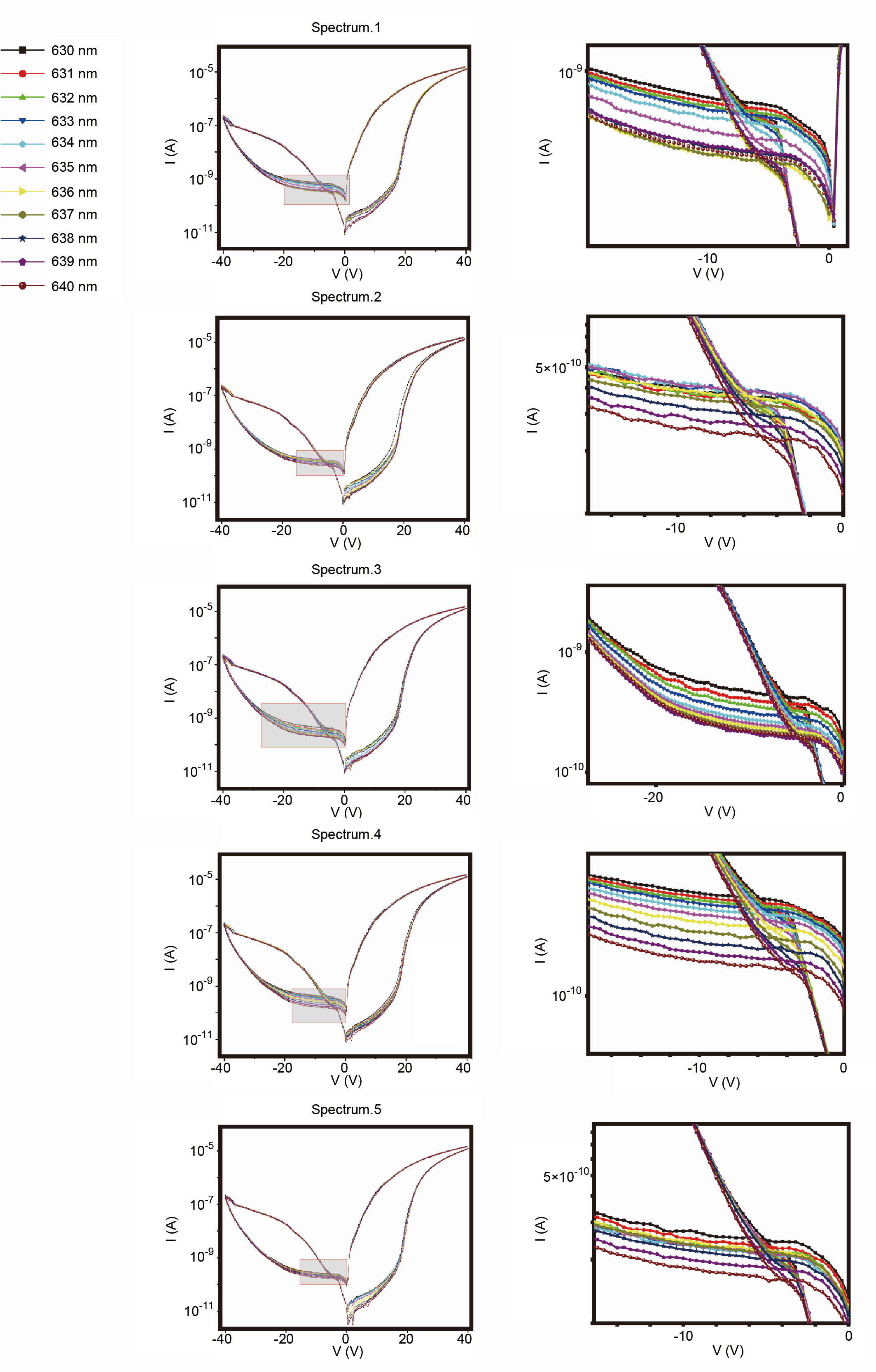


**Fig. S5:** **Diverse Photocurrent Responses across Wavelengths for Spectral 1 to Spectrum 5.** This figure showcases photocurrent datasets from Spectrum 1 to Spectrum 5, capturing the device's response to back-and-forth voltage scanning at 40 V across different wavelengths, specifically from 630 nm to 640 nm with a half-width of 2 nm. The lower panels offer an expanded view of specific regions, highlighted by red boxes in the upper panels, providing detailed insights into the photocurrent variations and demonstrating the device's sensitivity and resolution capabilities at these narrowly defined wavelength intervals.


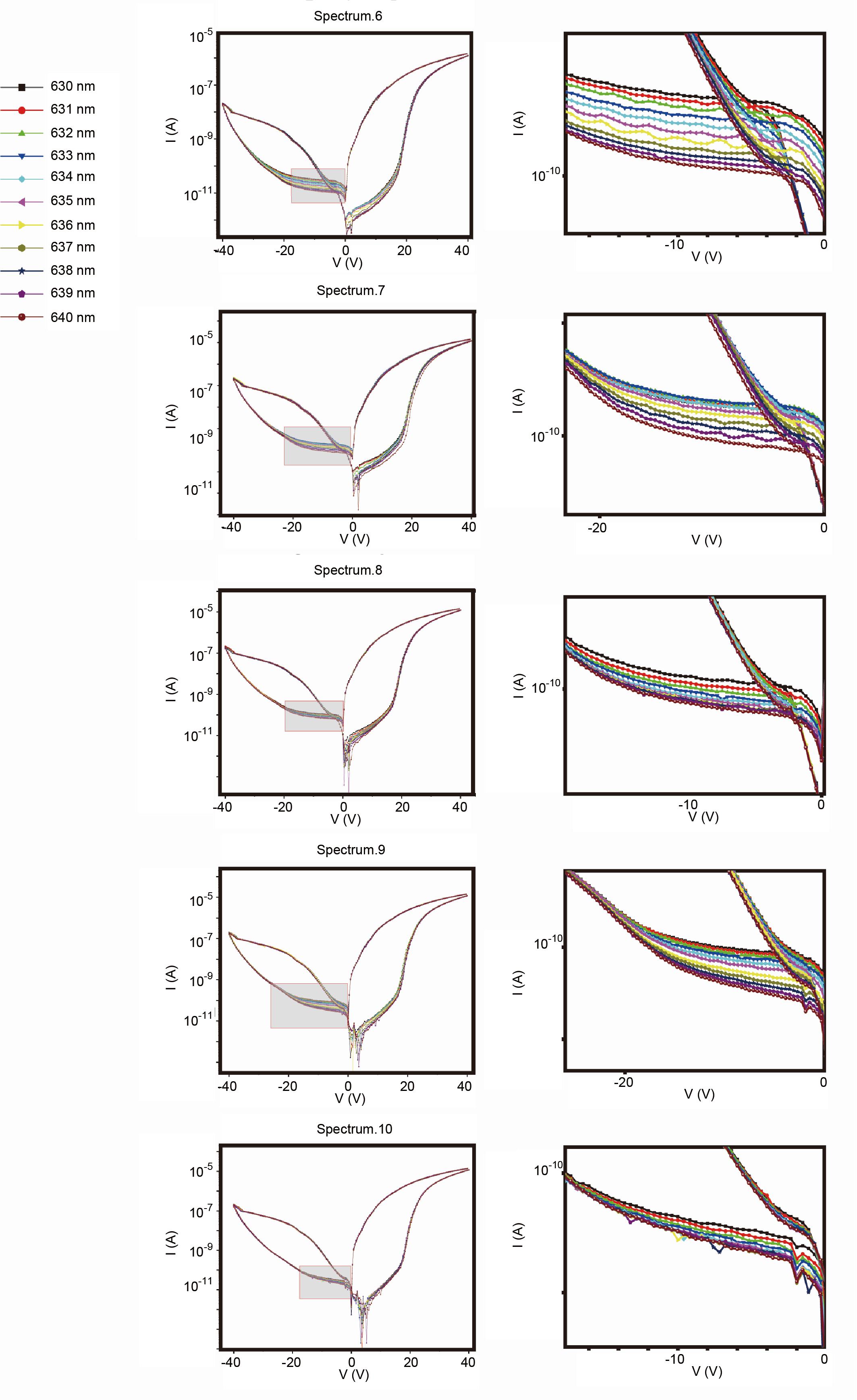


**Fig. S6. Diverse Photocurrent Responses across Wavelengths for Spectral 6 to Spectrum 10.** This figure presents the device's response to voltage scanning (40 V, back-and-forth) over different wavelengths, spanning 630 nm to 640 nm with a 2 nm half-width. Enhanced views of select areas, marked by red boxes in the upper panels, are displayed in the lower panels, offering detailed examination of photocurrent fluctuations and showcasing the device's acute wavelength resolution and sensitivity within this narrow wavelength range.


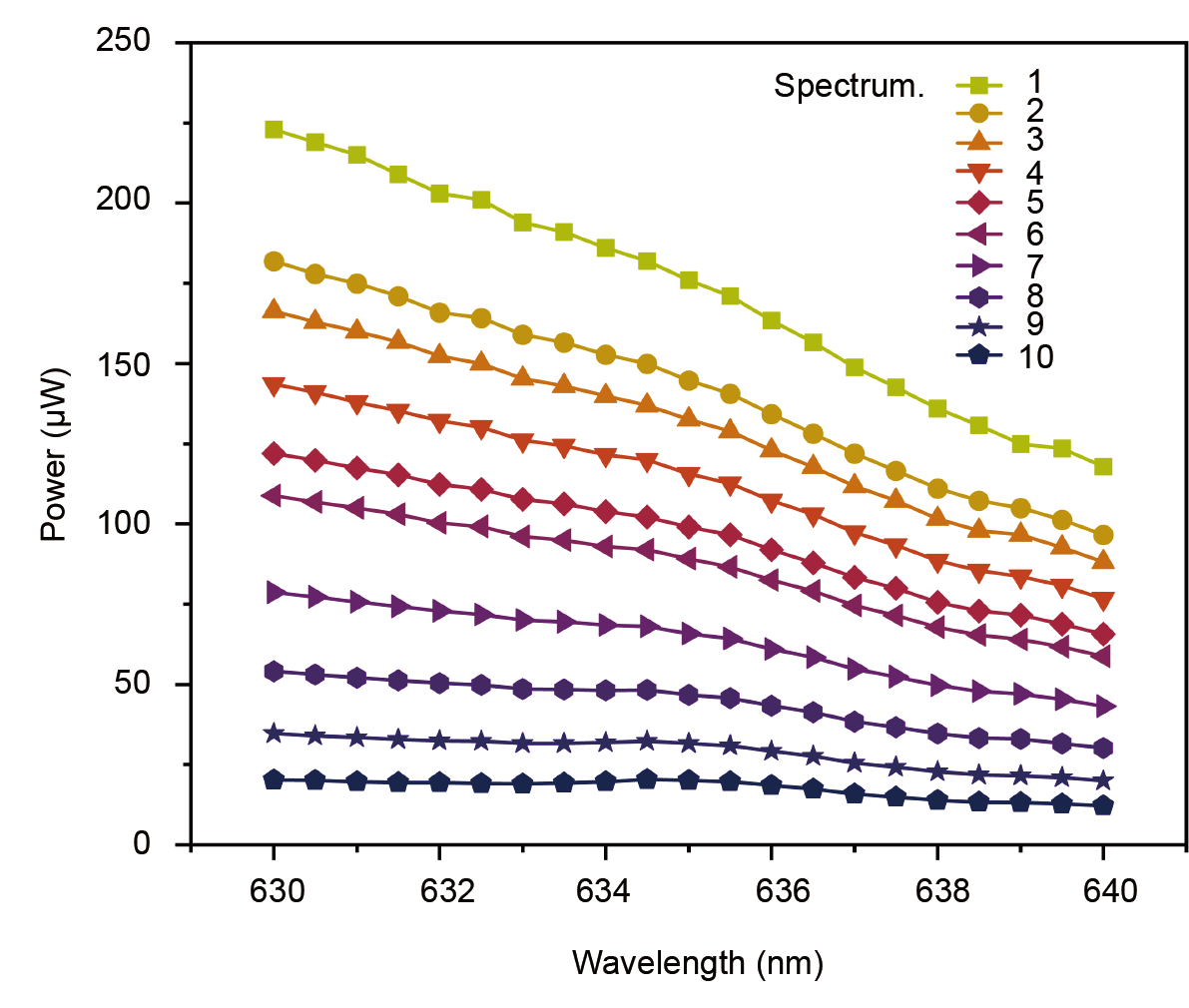


**Fig. S7. Measured spectral power for Spectrum 1 to Spectrum 10.** This figure displays the measured spectral powers for Spectrum 1 through Spectrum 10, with each curve representing the spectral power of the white source post-monochromatic filtering. These measurements reveal the differential power distribution across the spectra, providing insights into the filtering efficacy and spectral characteristics of the device under varying light conditions.


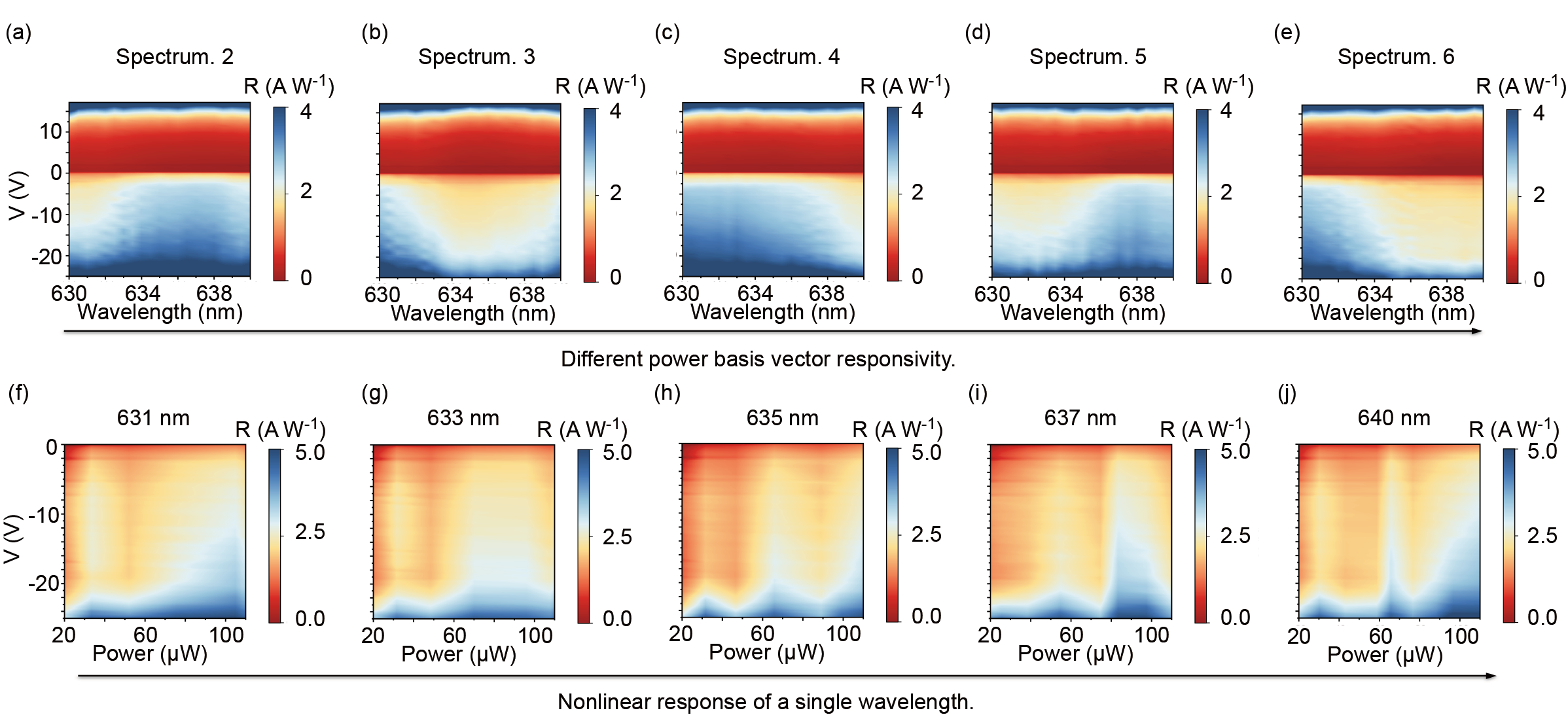


**Fig. S8:** **Partial Dynamic Nonlinear Response of the Memristor Across Power, Voltage, and Wavelengths.** This figure illustrates the memristor's nonlinear response as a function of varying parameters such as power, voltage, and wavelengths. Changes in voltage alter the device's responsivity due to dynamic shifts in the energy band structure. Increased power introduces a delay in the internal migration of Pd ions, significantly affecting the device's responsivity. This visualization underscores the intricate relationship between electrical input and the memristor's photonic output, highlighting its complex internal mechanisms.


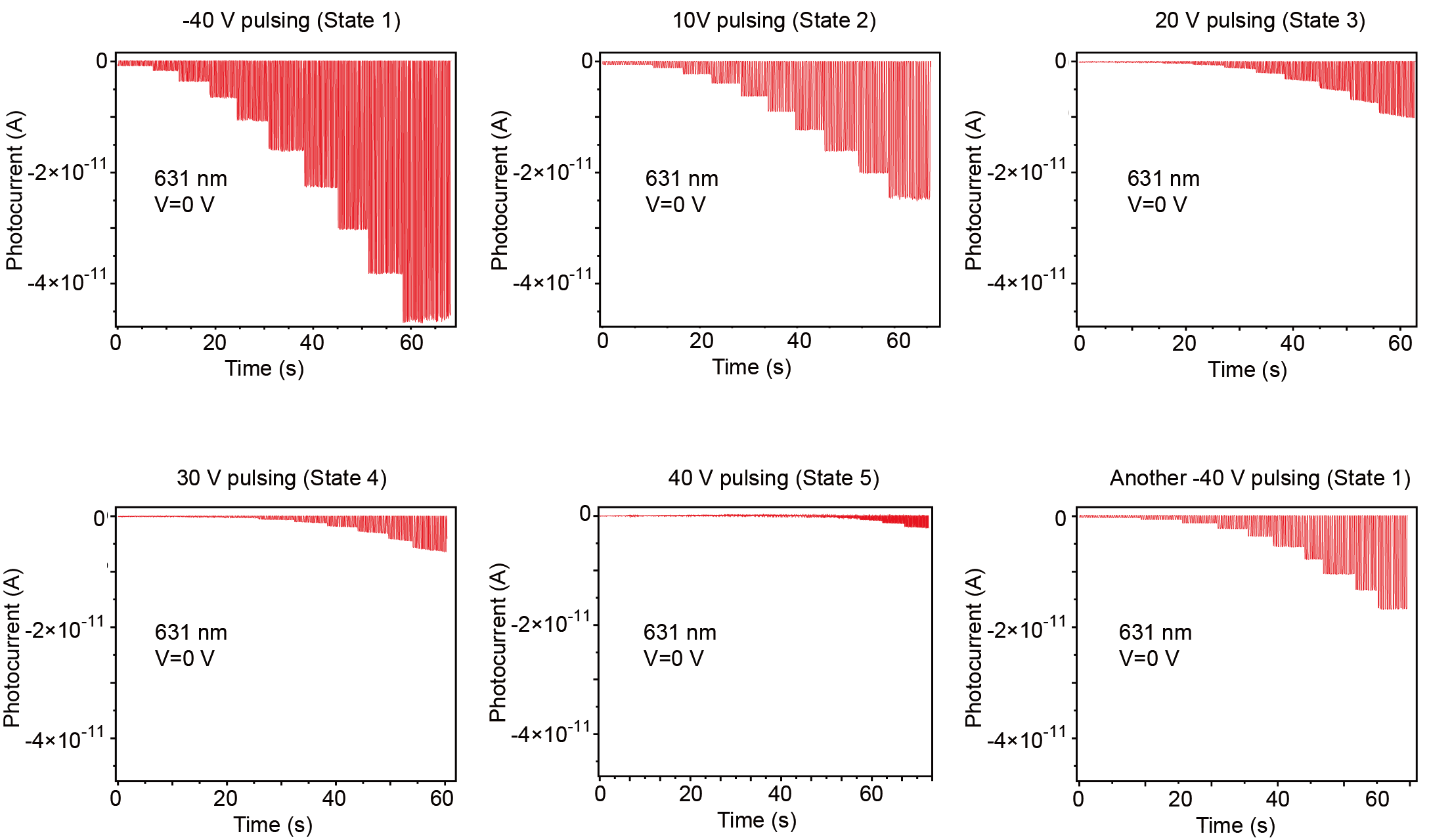


**Fig. S9. Nonlinear Response of Memristor under 631nm Irradiation.** State 1: -40 V, State 2: 10 V, State 3: 20 V, State 4: 30 V, and State 5: 40 V pulsing.


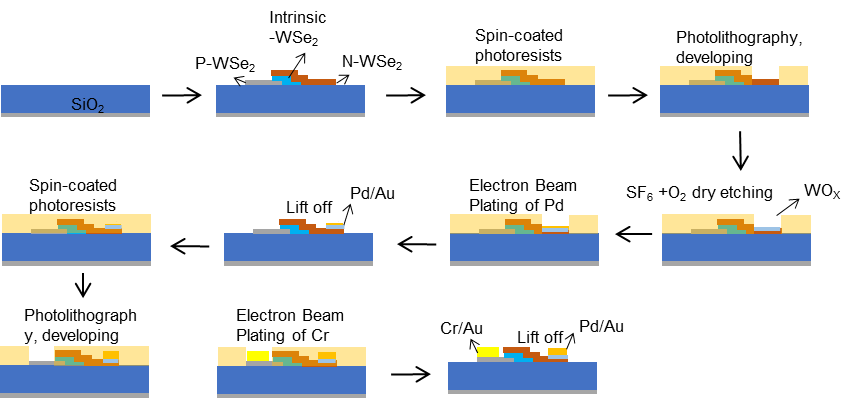


**Fig. S10: Process for Altered Memristive Behavior in PIN Devices.** This figure outlines the modified fabrication steps designed to suppress memristive behavior in the PIN device. Starting with the mechanical exfoliation of WSe_2_ flakes onto a SiO_2_/Si substrate, the process includes coating with photoresist and defining the anode pattern via laser lithography. The exposed regions are etched using SF_6_ and O_2_ gases at 150 W to prepare for electrode formation. Subsequently, Pd is deposited at a low speed to form the anode, followed by detachment (30 nm). The cathode end is similarly created using spin-coating, lithography, and detachment, finishing with Cr/Au deposition (5 nm/25 nm), aiming for a structure without inherent memristive characteristics.


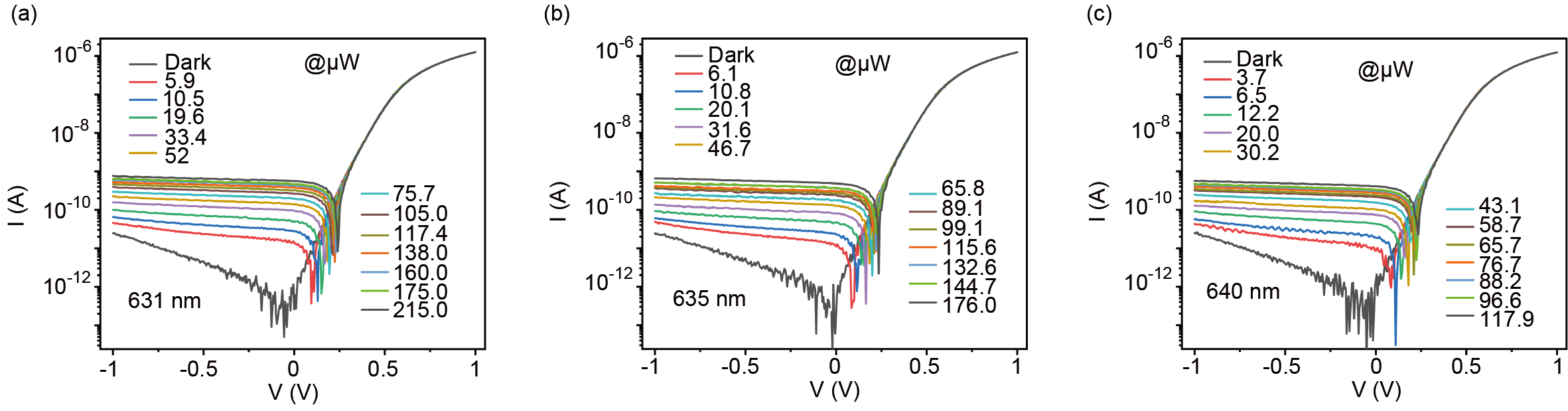


**Fig. S11: Photo- and Dark- Current Response in Non-Memristive Devices under Monochromatic Light.** This figure presents the photocurrent and dark current behavior of non-memristor devices stimulated with 631 nm (a), 635 nm (b), and 640 nm (c) monochromatic light. It is observed that these devices exhibit robust pn junction characteristics, with photocurrents increasing almost linearly in response to rising optical power. This linear increase in photocurrent, alongside a corresponding rise in photo-generated electromotive force, underscores the distinct electrical behavior of non-memristive devices under varying light conditions.


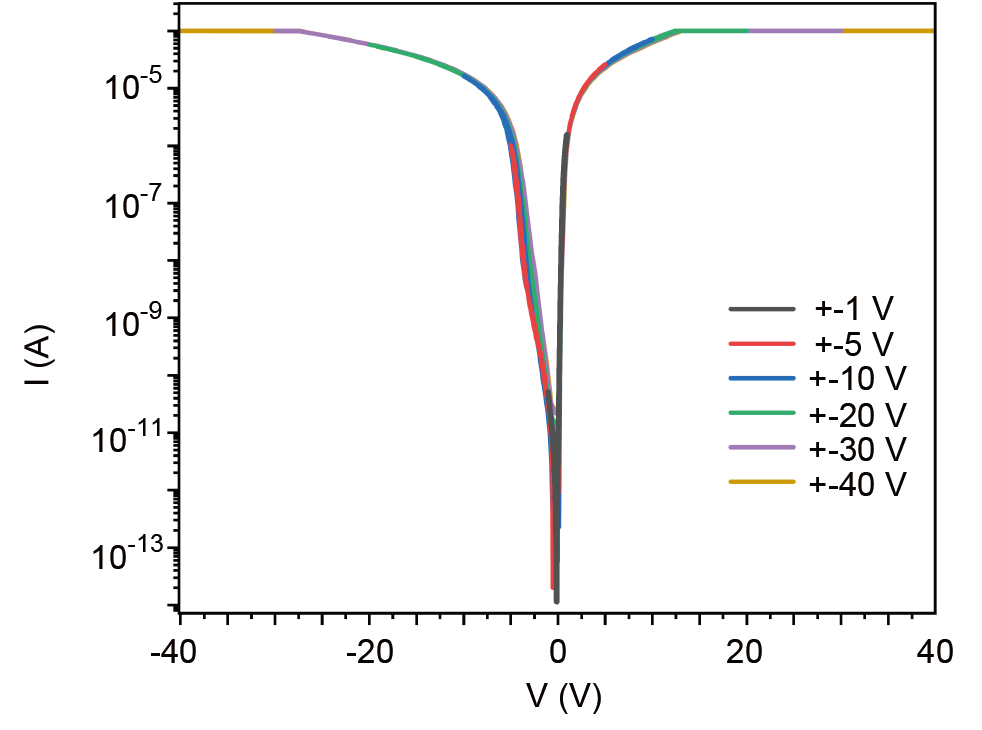


**Fig. S12: Output Characteristic Curves of Non-Memristor under Voltage Scanning.** This figure illustrates the output characteristic curves of a non-memristor device as the voltage is scanned from -40 V to +40 V. Unlike memristive counterparts, this device exhibits no memristive effects, serving as a stark contrast for intuitive comparison. The absence of memristive behavior is attributed to the lack of Pd ion implantation, preventing the dynamic modulation of the energy band and highlighting the critical role of ion migration in memristive functionality.


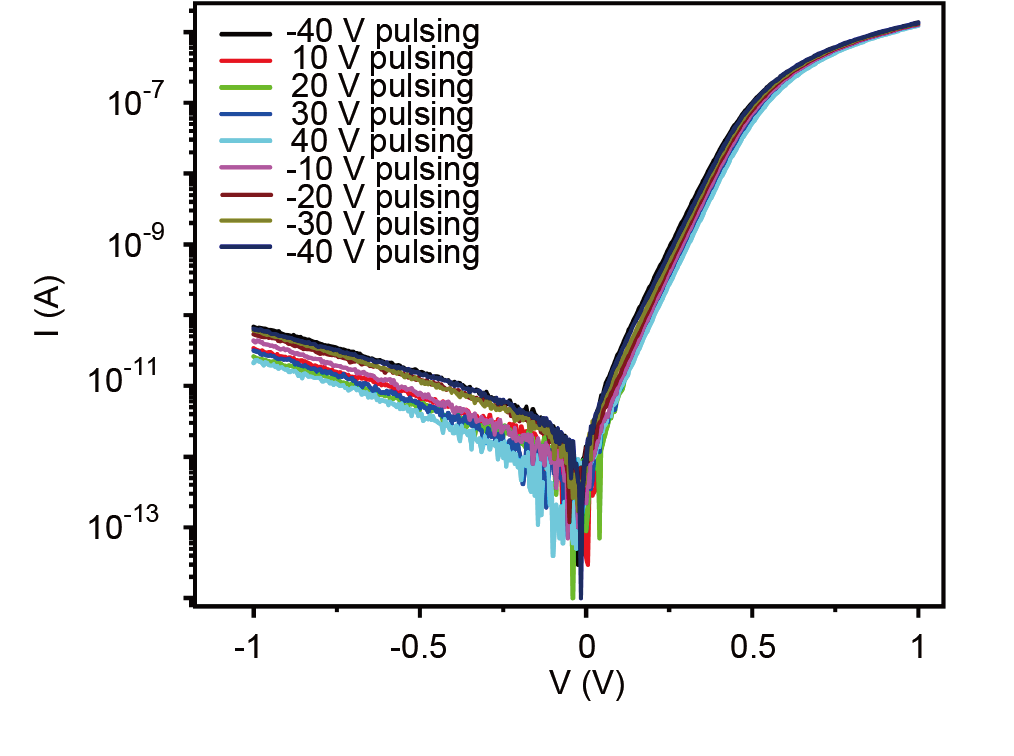


**Fig. S13:** **Dark Current Behavior in Non-Memristor Devices across Various States.** This figure examines the dark current characteristics of non-memristor devices, highlighting that their low-voltage polarity characteristics cannot be modulated in the same manner as memristors. Moreover, these devices lack the non-volatile nature inherent to memristors, emphasizing a significant distinction in their electrical behavior and memory retention capabilities.


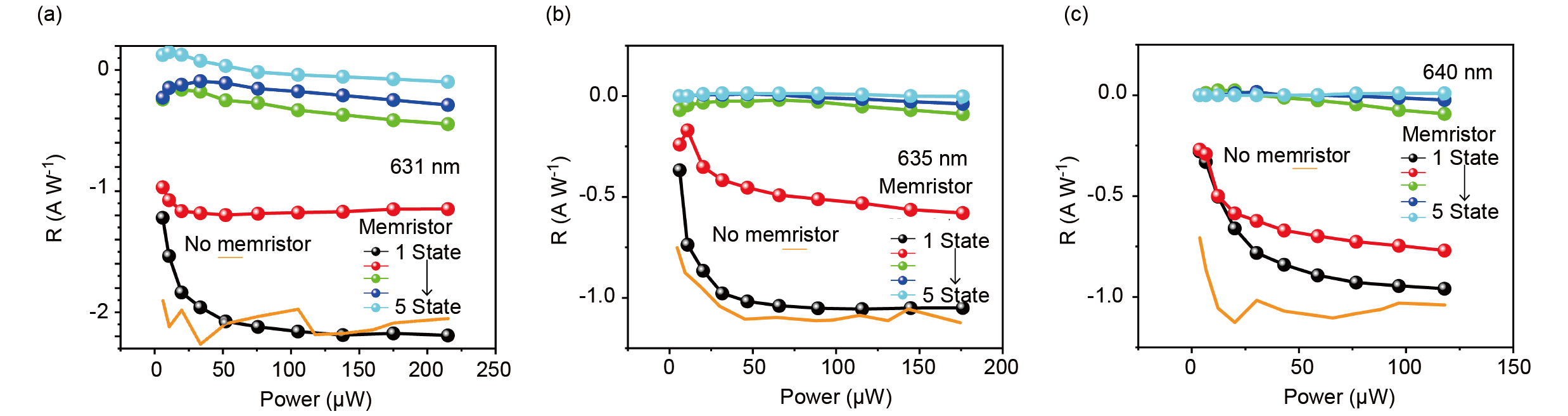


**Fig. S14: Responsivity Comparison between Memristor and Non-Memristor Devices.** This figure contrasts the responsivity of memristor and non-memristor devices under optical stimuli of 631 nm (a), 635 nm (b), and 640 nm (c) across different states (State 1: -40 V, State 2: 10 V, State 3: 20 V, State 4: 30 V, and State 5: 40 V pulsing). The comparison delineates the distinctive response behaviors, highlighting the memristor's advanced functionality and the non-memristor's static response under identical conditions.


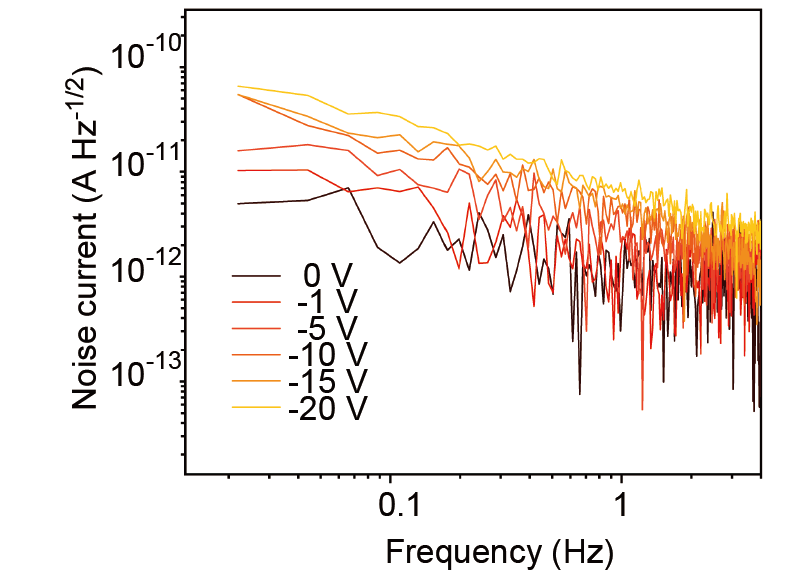


**Fig. S15: Noise currents at different operating voltages of the device.** Noise currents at different operating voltages of the device. The low noise current is crucial for achieving low noise equivalent power. These results demonstrate the feasibility of using the Pd ion migration mechanism for the spectral reconstruction of weak light.


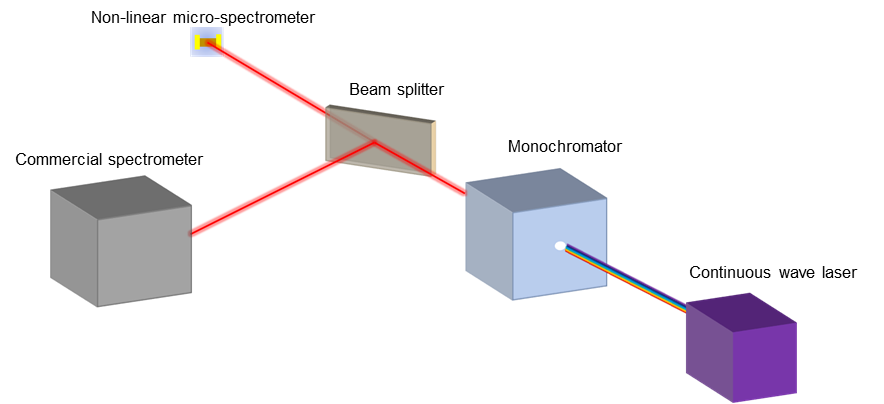


**Fig. S16:** **Spectrometer Measurement Optical Setup.** This figure presents a schematic of the optical setup utilized for reconstructing unknown spectra using our single-junction photonic memristor. A white light source emits a continuous spectrum, processed by a monochromator to yield monochromatic, narrow-bandwidth light. This light is then split; one path directs light towards the nonlinear memristor for analysis, while another path leads some light to a commercial spectrometer for reference measurements, illustrating the comprehensive approach to spectral data collection and analysis.


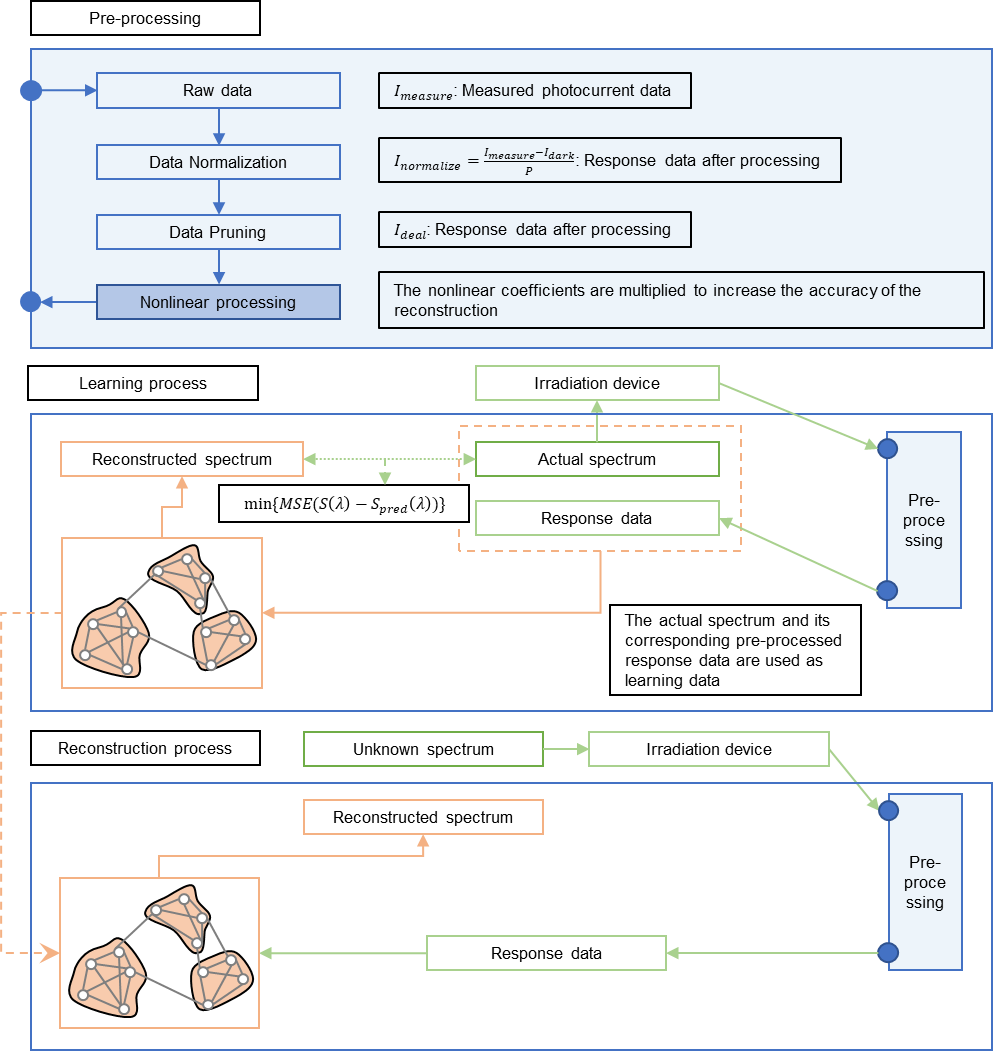


**Fig. S17:** **Spectral Reconstruction via Nonlinear Neural Networks.** This figure outlines the spectral reconstruction process using nonlinear neural networks, tailored to accommodate the device's nonlinearity which renders traditional methods ineffective. It details the initial data preprocessing to derive usable optical response data, followed by the neural network's training phase aimed at minimizing the mean squared error (MSE) between the reconstructed and actual spectra, with Adam chosen as the optimizer. As training progresses, the learning rate decreases. The final step involves using the trained network to predict unknown spectra, incorporating nonlinear processing for varying power levels or complex spectral incidences, thus enhancing the network's adaptability to diverse spectral inputs.


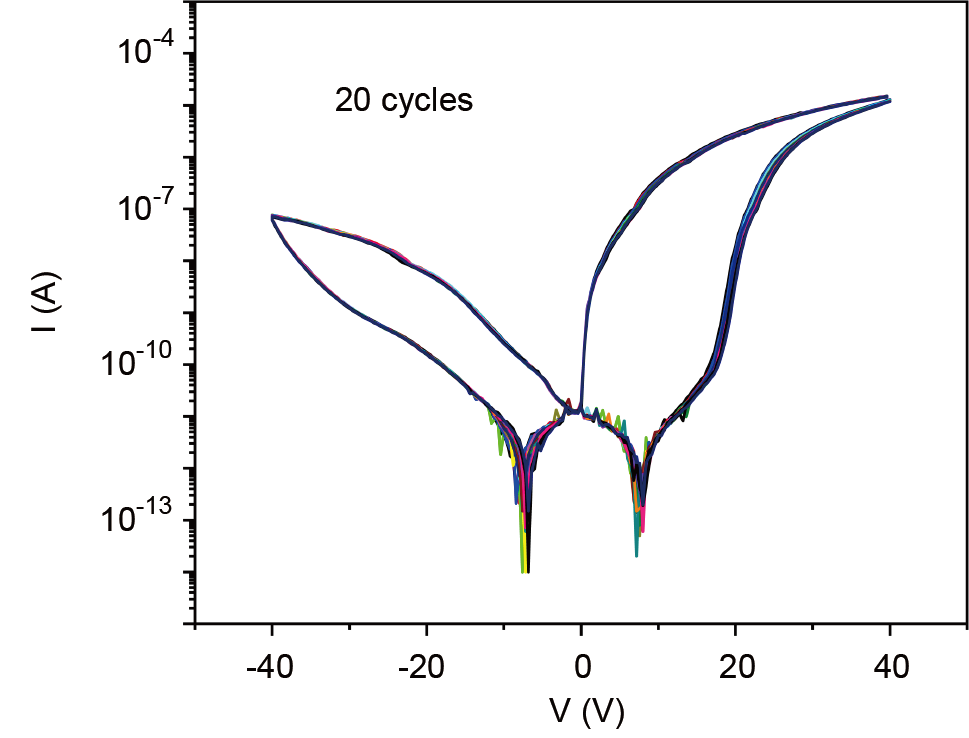


**Fig. S18: Dark current repeatability property of the fabricated memristors.** This figure displays the outcomes of scanning the memristor's dark current 20 times, demonstrating high repeatability. Such consistency is crucial for the accuracy of spectral prediction, underscoring the device's reliability and the predictability of its performance in spectral analysis tasks.


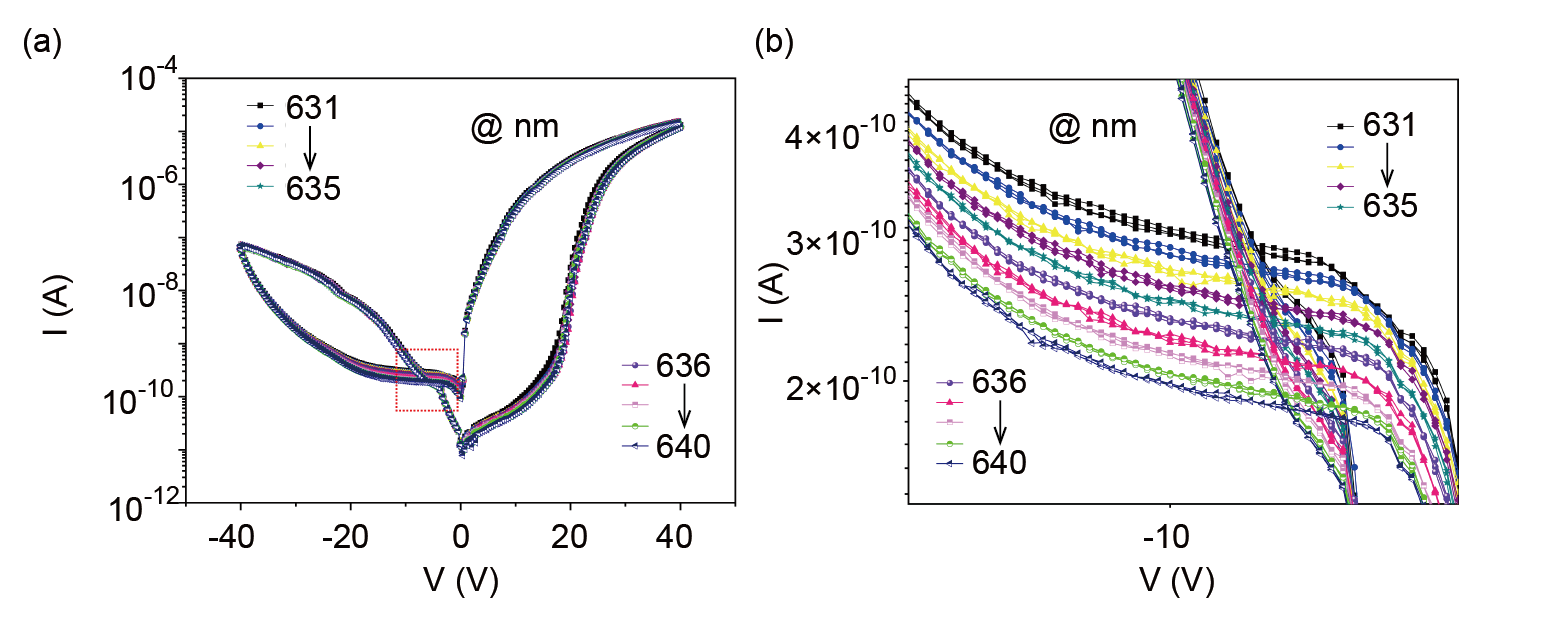


**Fig. S19.** **Photocurrent repeatability of the fabricated memristors.** This figure highlights the results from three consecutive photocurrent scans under identical conditions, showcasing negligible differences between the trials. The near-overlapping results under the same experimental setup affirm the memristor's operational consistency. This repeatability indicates that the device's testing variance falls well below its accuracy threshold, thereby validating the reliability of the memristor for precise spectral analysis applications.


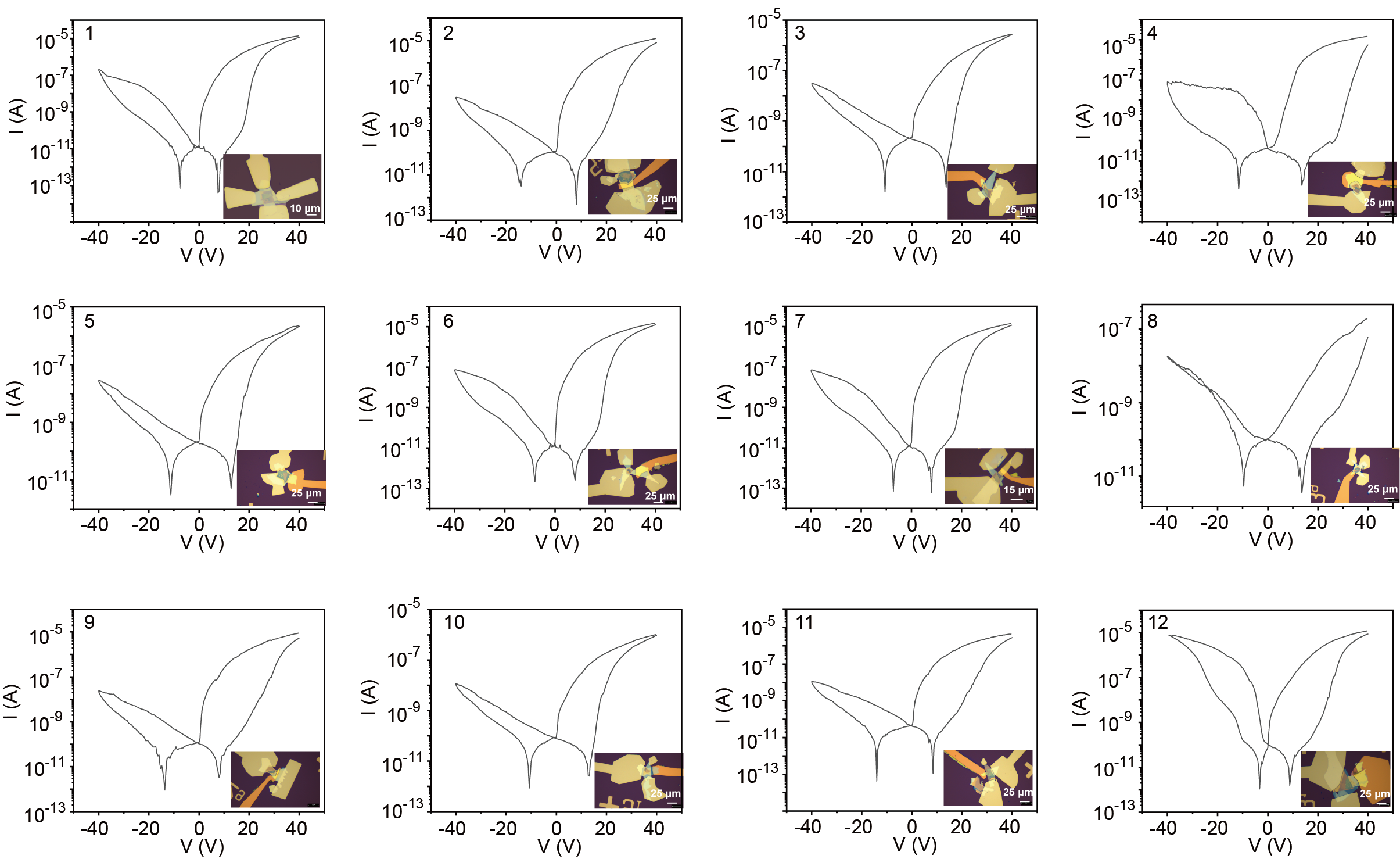


**Fig. S20. Dark** **IV curves of the** **fabricated multiple devices.** It includes 11 newly fabricated devices. Device #1, used in the main text acts as a reference.

The following outlines the fabrication details, performance metrics, and potential methods for improving consistency:

**Fabrication Process:**

We sequentially transferred n-type WSe_2_, intrinsic-WSe_2_, and p-type WSe_2_ onto Si/SiO_2_ substrates. The Pd electrode was deposited at a vaporization rate of 0.5 Å s^-1^, with the sample plate rotating at 20 rpm to ensure uniform coating across the devices. The thickness of the Pd electrodes was 30 nm, consistent with the device process flow described in the manuscript. Subsequently, Cr/Au electrodes with a thickness of 5/25 nm were evaporated .

**Performance Metrics and Analysis:**

We tested the dark current characteristics of the 11 newly fabricated devices, comparing their performance with that of the device (#1) used in the manuscript. As shown in Fig. S20, all devices exhibited good contact and the characteristic "butterfly" hysteresis, indicative of the memristive behavior. The magnitude of the dark current at the same voltage was consistent across most devices, with only minor variations observed.

Upon analysis, we identified that these variations were primarily due to differences in the thicknesses and dimensional sizes of the materials used in the mechanical exfoliation process. For instance, devices with larger junction areas, such as #2, #4, and #12, exhibited higher currents. Conversely, smaller device sizes and thinner layers led to weaker memristive characteristics, as observed in device #8.

**Mitigating Variations and Improving Reproducibility:**

To further enhance the reproducibility of the devices, we recommend the following approaches:

**a. Uniform Growth Methods:** While the mechanical exfoliation process demonstrates good repeatability, absolute reproducibility can be further improved by employing large-area uniform growth techniques, such as wafer-level chemical vapor deposition (CVD).

**b. Precision Cutting:** Implementing precision cutting methods after fabrication can significantly enhance device uniformity and repeatability.

**c. Individual Calibration:** Each device should be individually calibrated to ensure a uniform response during subsequent use.

**d. Standardized Pre-Processing:** Uniform pre-processing of device data, such as normalization of each data set, can reduce errors and improve the consistency of measurements.


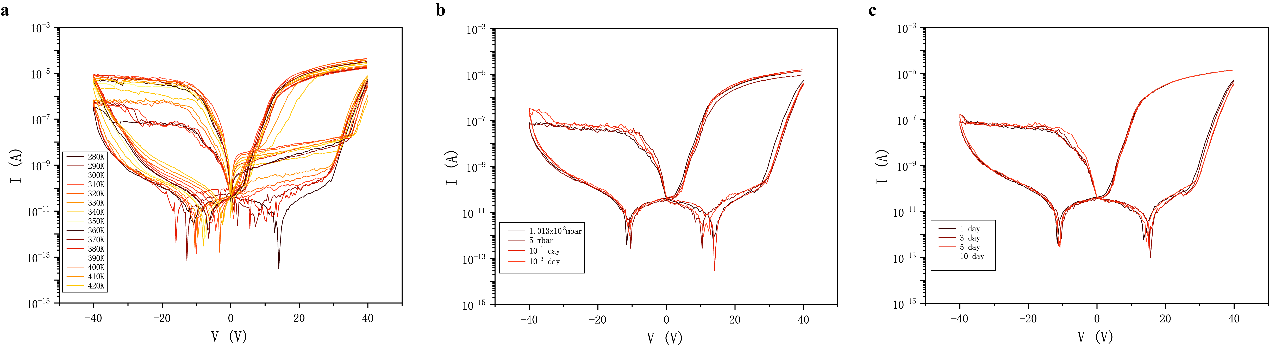


**Fig. S21. The environment robustness evaluation of the fabricated device.** (a) Dark current of the device at different temperatures. (b)Dark current of the device under pressure 1.013 × 10^3^ mbar, 5 mbar, 10^-1^ mbar, and 10^-3^ mbar, respectively. (c) The dark current of the device at 1, 3, 5, and 10 days.


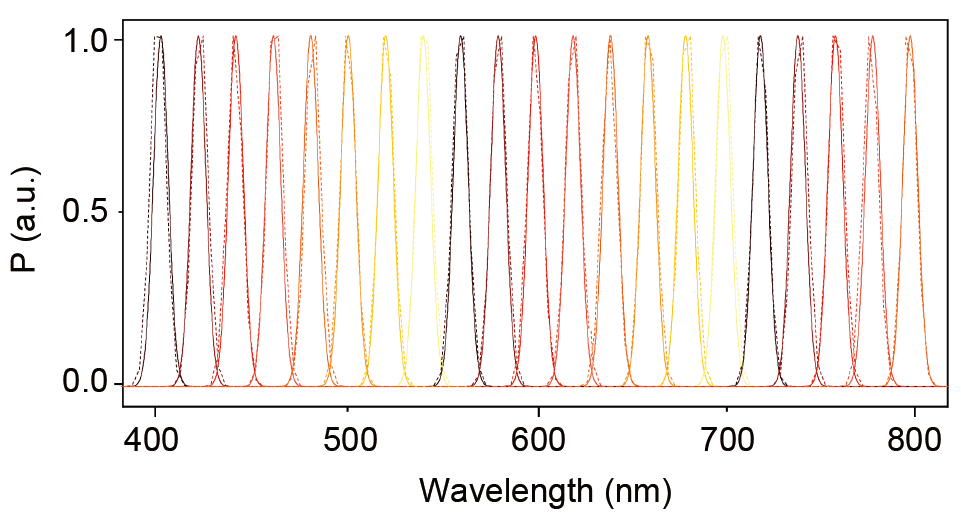


**Fig. S22. Reconstruction of single peak spectrum with narrow bandwidth across 400-800 nm.** Spectral reconstruction with 20 nm intervals and a half-height width of 4 nm, across the WSe_2_ response spectrum in the 400-800 nm range.


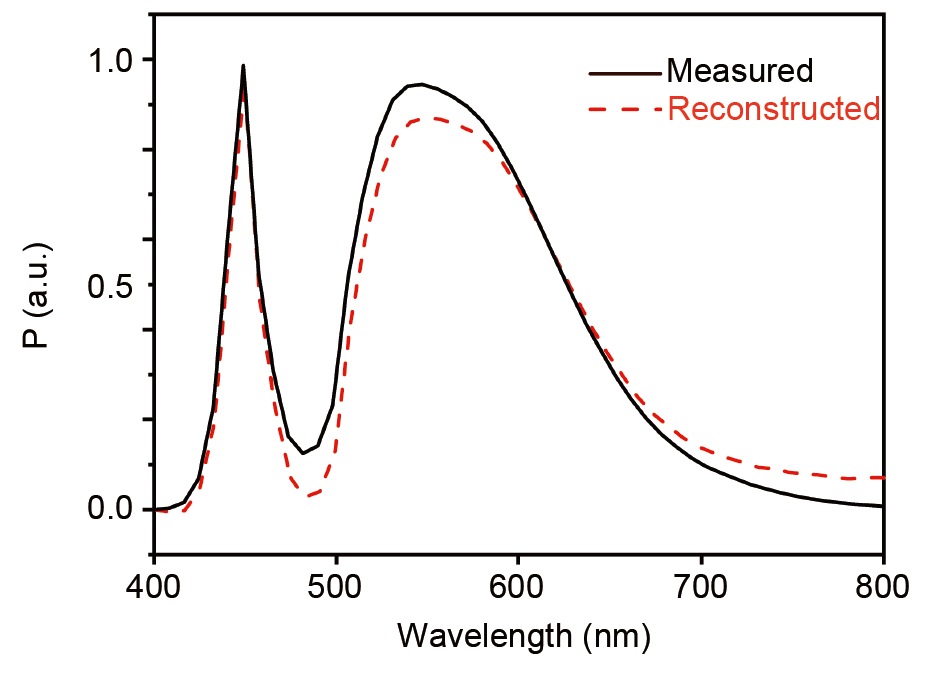


**Fig. S23.** **Reconstructed versus actual spectra for large bandwidth across 400nm-800nm.** We reconstructed the 400-800 nm spectra based on this algorithm, and it can be seen that the reconstructed spectra are basically consistent with the input spectra.

**Table S1: Performance comparison of reported computational spectrometers and ours.**

| **Reference** | **Physics or method (material or device)** | **Structure or**  **requirement** | **Footprint** | **Spectral**  **resolution** | **Spectral**  **range (nm)** | **wavelength accuracy** |
| --- | --- | --- | --- | --- | --- | --- |
| **(4)** | **Nanowire bandgap gradation (single CdS_x_Se_1−x_ nanowire)** | **Detector array** | **75×0.5 μm^2^** | **~10nm** | **500-630** | **/** |
| **(5)** | **Bandgap tuned by Stark effect (black phosphorus channel)** | **Single detector @80K** | **16×9 μm^2^** | **~90nm** | **2000-9000** | **/** |
| **(6)** | **Tunable quantum efficiency (superconducting nanowire)** | **Single detector @1.65K** | **6×6 μm^2^** | **~6nm** | **660-1900** | **/** |
| **(7)** | **Tunable interlayer excited state transition (ReS_2_/Au/WSe_2_ vdW heterostructure)** | **Single detector @80K** | **6×4 μm^2^** | **~20nm** | **1150-1470** | **/** |
| **(8)** | **Tunable interlayer transport (MoS_2_/WSe_2_ vdW heterojunction)** | **Single detector under**  **ambient conditions** | **22×8 μm^2^** | **~3nm** | **405-845** | **0.36 nm** |
| **(9)** | **Tunable interlayer transport (MoS_2_/BP vdW heterojunction)** | **Single detector @300K** | **30×20 μm^2^** | **~2nm** | **1700-3600** | **/** |
| **(10)** | **Tunable interlayer transport (SnS_2_/ReSe_2_ vdW heterojunction)** | **Single detector @300K** | **22×10 μm^2^** | **~5nm** | **400-800** | **/** |
| **This work** | **Our memristive energy band regulation (P-I-N WSe_2_ vdW heterojunction)** | **Single detector @300K** | **4×****9 μm^2^** | **~2nm** | **400-800** | **0.16 nm** |

**References**

- - 1. Liu, E. et al. Signatures of moiré trions in WSe_2_/MoSe_2_ heterobilayers. *Nature* 594, 46-50 (2021).
    2. Chen, Y. et al. Unipolar barrier photodetectors based on van der Waals heterostructures. Nature. Electronics 4, 357-363 (2021).
    3. Wang, Z. et al. The ambipolar transport behavior of WSe_2_ transistors and its analogue circuits. NPG Asia Materials 10, 703-712 (2018).
    4. Yang, Z. et al. Single-nanowire spectrometers. Science 365, 1017-1020 (2019).
    5. Yuan, S. et al. A wavelength-scale black phosphorus spectrometer. Nat. Photonics 15, 601-607 (2021).
    6. Kong, L. et al. Single-detector spectrometer using a superconducting nanowire. Nano Lett. 21, 9625-9632 (2021).
    7. Deng, W. et al. Electrically tunable two-dimensional heterojunctions for miniaturized near-infrared spectrometers. Nat. Commun. 13, 4627 (2022).
    8. Hoon, H. Y. et al. Miniaturized spectrometers with a tunable van der Waals junction. Science 378, 296-299 (2022).
    9. Uddin, M. G. et al. Broadband miniaturized spectrometers with a van der Waals tunnel diode. Nature Communications 15, 571 (2024).
    10. Wu, G. et al. Miniaturized spectrometer with intrinsic long-term image memory. Nature Communications 15, 676 (2024).
